# 3D printed biaxial stretcher compatible with live fluorescence microscopy

**DOI:** 10.1016/j.ohx.2020.e00095

**Published:** 2020-01-21

**Authors:** Daniel J. Shiwarski, Joshua W. Tashman, Amity F. Eaton, Gerard Apodaca, Adam W. Feinberg

**Affiliations:** a Department of Biomedical Engineering, Carnegie Mellon University, Pittsburgh, PA, United States; b Department of Materials Science & Engineering, Carnegie Mellon University, Pittsburgh, PA, United States; c Department of Medicine, Renal-Electrolyte Division, and Cell Biology, University of Pittsburgh, Pittsburgh, PA United States

**Keywords:** Cell stretcher, Fluorescence imaging, Mechanobiology, Tensile testing, Tissue mechanics, Biaxial strain

## Abstract

Mechanical characterization and tensile testing of biological samples is important when determining the material properties of a tissue; however, performing tensile testing and tissue stretching while monitoring cellular changes via fluorescence microscopy is often challenging. Additionally, commercially available cell/tissue stretchers are often expensive, hard to customize, and limited in their fluorescence imaging compatibility. We have developed a 3D printed Open source Biaxial Stretcher (OBS) to be a low-cost stage top mountable biaxial stretching system for use with live cell fluorescence microscopy in both upright and inverted microscope configurations. Our OBS takes advantage of readily available open source desktop 3D printer hardware and software to deliver a fully motorized high precision (10 ± 0.5 µm movement accuracy) low cost biaxial stretching device capable of 4.5 cm of XY travel with a touch screen control panel, and an integrated heated platform with sample bath to maintain cell and tissue viability. Further, we designed a series of tissue mounts and clamps to accommodate varying samples from synthetic materials to biological tissue. By creating a low-profile design, we can directly mount the stretcher onto a microscope stage, and through coordinated biaxial stretching we maintain a constant field of view facilitating real-time sample tracking and time-lapse fluorescence imaging.

## Graphical abstract

**Figure:**
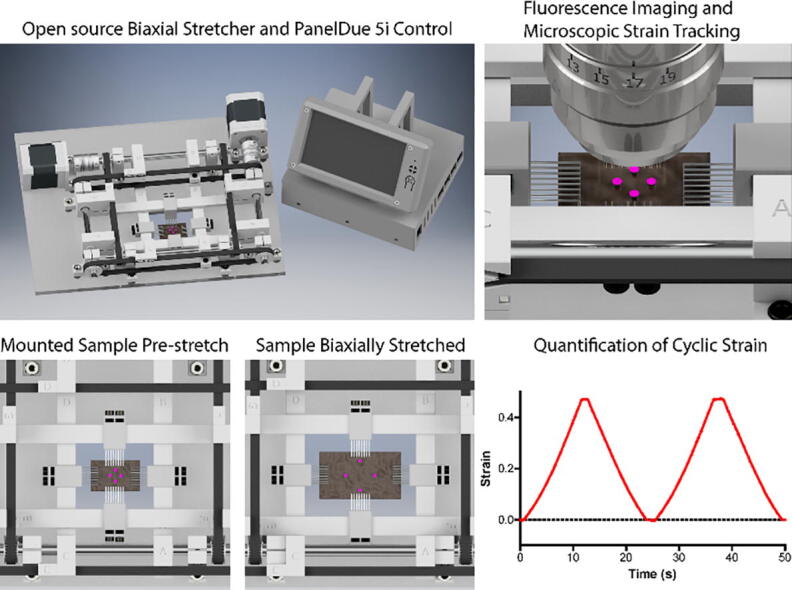

**Specifications Table:** 

Hardware name	Open source Biaxial Stretcher (OBS)
Subject area	• Engineering and Material Science • Biological Sciences (e.g. Microbiology and Biochemistry)
Hardware type	• Imaging tools • Measuring physical properties and in-lab sensors • Biological sample handling and preparation
Open Source License	CC-BY 4.0
Cost of Hardware	*$580*
Source File Repository	https://doi.org/10.5281/zenodo.3483849

## 1 Hardware in context

### 1.1 Introduction

Mechanical forces play an important role in tissue development, organ function, and disease pathology and have spurred the development of new methods to study cellular and tissue biomechanics [1], [2], [3], [4], [5], [6]. There are many ways to characterize the biomechanical properties of tissues, including uniaxial tensile testing, compression testing, indentation testing, and atomic force microscopy [7], [8], [9]. However, there are a limited number of approaches that are capable of uniaxial or biaxial testing of cells and/or tissue while simultaneously monitoring cellular changes in morphology and behavior [6]. The commercial devices that do exist are often expensive (>$20,000), require specialized software, and do not allow for live fluorescence imaging capabilities in combination with environmental temperature control, which is often an experimental necessity [5], [6].

**Fig. 1:**
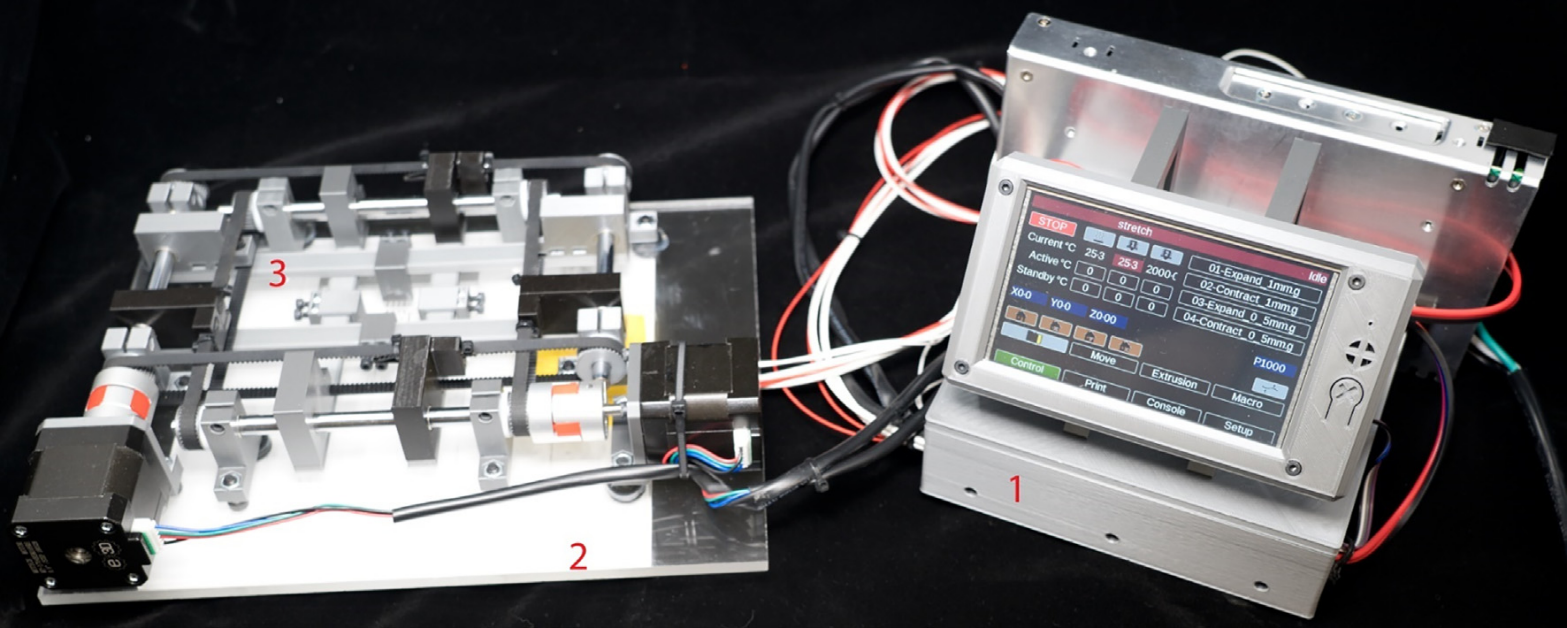
The 3D printed open source biaxial stretcher (OBS) with PanelDue 5i touch display and duet WiFi control board. (1) The Duet WiFi control box. (2) The heated bed plate. (3) The biaxial stretcher and sample mounts.

Here we report development of an open source biaxial stretcher (OBS), which was designed to be a low-cost stage top mountable biaxial stretching system for use with live cell fluorescence microscopy in both upright and inverted microscope configurations. Our goals in the creation of the OBS were to: (1) utilize open source 3D printer hardware, software, and electronics for construction of a low cost biaxial stretching device, (2) create a low profile design capable of direct mounting on top of a microscope stage, (3) maintain a constant field of view when performing biaxial stretching for effective sample tracking and time lapse imaging, and (4) design submersible, removable, tissue mounts and clamps to accommodate varying sample types and sizes. The result is a customizable, inexpensive, 3D-printable biaxial stretcher capable of 4.5 cm of XY travel, with an integrated heated platform to maintain cell and tissue viability (Fig. 1). Full computerized control is provided by a Duet WiFi motion control board that is interfaced with a PanelDue 5i touch display, WiFi cell phone application, or direct USB connection. In addition to microscopy-based imaging applications, the OBS can be used in combination with a traditional camera-based imaging system to track material deformation and strain using fiducial markers following biaxial or uniaxial stretch.

### 1.2 Cost

Commercial biaxial stretchers, while high performance devices, can cost between $20,000 and $100,000 (e.g., Strex, CellScale) depending on the capabilities and configurations one chooses. They are also limited in their customization due to precision machined components and a mix of proprietary hardware and software that can be difficult to adapt to new use cases for which they were not originally designed. Several research groups have designed their own uniaxial or biaxial stretching devices for mechanical characterization of soft materials and biological specimens [5], [6], but no attempts have been made to specifically design a 3D printed biaxial stretching device for the characterization of live tissue samples via live cell fluorescence microscopy, and with precise mechanical performance.

To address this issue of cost, design flexibility, and performance, we utilized desktop 3D printing technology and readily available 3D printer-based hardware and electronics to construct the high-performance OBS for $580 (see *Bill of Materials*). With the addition of 3D printed interchangeable tissue clamps and rakes to accommodate a range of samples, the OBS system offers a unique opportunity for researchers to implement a customizable biaxial stretching system into their experimental workflow. Most of the parts were printed using a Prusa i3 MK3 desktop 3D printer, and the remaining components such as the Duet WiFi control board, PanelDue 5i screen, power supply, steel rods, nuts, bolts, and belts were purchased from online retailers.

### 1.3 Biaxial stretching of biological samples

Most tissues in the human body experience complex physiological loading in multiple axes, making simple uniaxial tensile testing inadequate for robust characterization of mechanical and biological properties [9]. Biaxial testing better recapitulates these loading regimes, but these systems are necessarily more complex than their uniaxial counterparts, which can often make them bulky. A more compact system such as our biaxial stretching system is required to combine mechanical loading with fluorescence microscopy applications, as the entire device must be able to fit upon or beneath the microscope system. It is also important for a region of interest to remain in the field of view and focal plane of the microscope objective during stretching for sample tracking and analysis. By stretching the sample symmetrically about the two orthogonal axes, as our biaxial stretcher system does, this condition can be met near the center of the sample. These design considerations make our biaxial stretcher system ideal for live cell and tissue fluorescence imaging.

### 1.4 Stretcher performance

The ability to biaxially stretch biological tissues and soft hydrogels combined with fluorescence microscopy, or an overhead camera, is a valuable research tool, but usually requires custom high-end hardware and software. By using a combination of open source and low-cost 3D printer software and hardware components, we can achieve uniaxial and biaxial strains of greater than 200% depending upon the sample shape, size, and elasticity. The low-profile design of the OBS enables integration with a range of upright and fluorescent microscope systems, such as the upright Nikon FN1 A1R MP+. We also have full control over strain rate, duration, periodicity, and ratio between X and Y strain, in a fully programmable manner. Lastly, the Duet WiFi 3D printer control board provides integrated heated sample control to maintain physiological temperature, or for the investigation of temperatures up to 150 °C for non-biologic samples. At $580, this system provides high-performance biaxial stretching at a fraction of the cost of commercial systems, but as an open source design provides an accessible platform to customize according to specific experimental requirements.

## 2 Hardware description

The OBS takes advantage of motion control and hardware developed for open source 3D printing to provide a compact, inexpensive, and capable biomechanical testing system that can be fabricated, assembled, and operated by following the included guide. Most components of the OBS are 3D printed, which lowers cost and allows for easy customization. All designs are licensed as open source under a CC BY-SA 4.0 license. The non-printed components consist of standard hardware such as bearings, linear motion rods, nuts and bolts, 3D printer belts, stepper motors and couplers, heaters, and the Duet WiFi and PanelDue 3D printer board and display. The OBS consists of three main assemblies shown in Fig. 1: (1) The Duet WiFi control box which houses the motion control and touch interface systems, (2) The heated bed plate which allows for heating samples, and (3) The biaxial stretcher which consists of the motorized biaxial motion platform and sample mounts. Individual subassemblies are shown as renders in the build instructions (Specifications Table).

### 2.1 The Duet WiFi control box

**Fig. 2:**
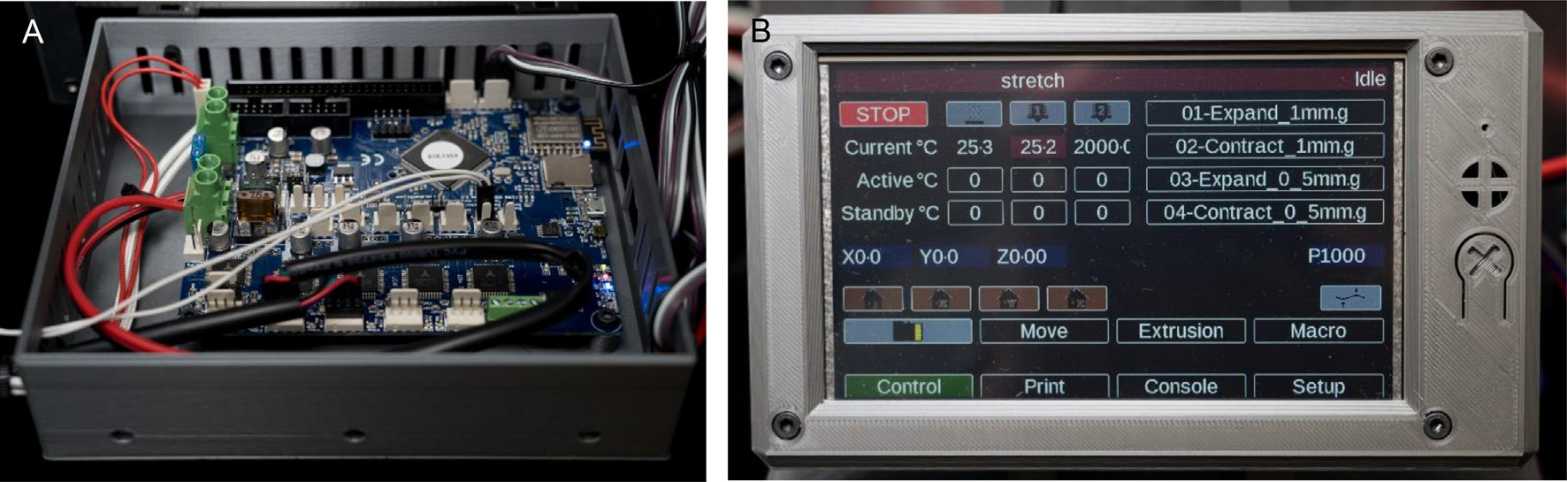
Duet WiFi and PanelDue 5i in 3D printed housing. (A) Duet WiFi board mounted in 3D printed enclosure. (B) PanelDue 5i mounted in 3D printed enclosure.

The OBS uses an open source motion control board designed for 3D printers called the Duet WiFi (Fig. 2A). This system runs on a 32 bit microprocessor and has integrated control of up to 5 stepper motors, two heaters with closed loop control, and multiple fans. Future integration of external sensors and hardware is available through an expansion header. The Duet WiFi is compatible with a plug and play color touchscreen interface (the PanelDue 5i, Fig. 2B) that allows the user to manually operate the biaxial stretcher or activate pre-programmed stretching protocols. Additionally, control is available through a direct USB connection to a computer or via the built-in web interface. The 3D printer numerical control programming language utilized for stretching is g-code, which can be customized for specific uniaxial or biaxial stretching protocols. The Duet WiFi comes pre-loaded with firmware and is fully operational out of the box. Firmware updates can be installed through the web server interface that the board comes pre-configured to host. All procedures related to modifying the operating parameters of the board can be found at https://duet3d.dozuki.com/. To house the Duet WiFi, a control box consisting of a 3D printed enclosure for the Duet WiFi with a lid that incorporates a mounting point for the Panel Due display and mounts for a 12 V switching power supply was designed.

### 2.2 The heated bed plate

**Fig. 3:**
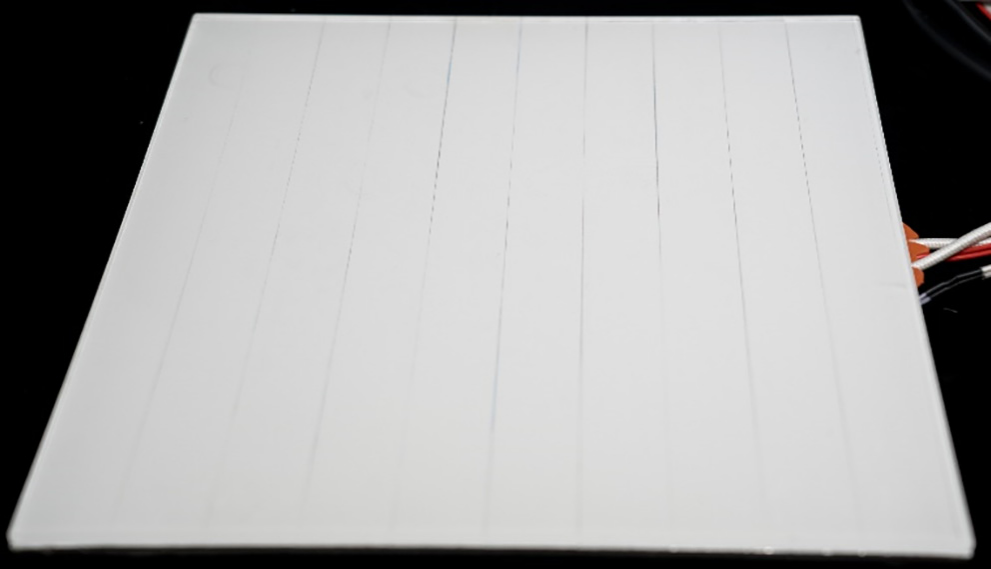
Picture of the assembled heated bed plate.

Biological samples typically need to be maintained at a physiological temperature of 37 °C during experiments to maintain viability and ensure that the properties measured are representative of normal physiology. To achieve this, the biaxial stretcher system incorporates a heated bed plate that consists of a silicone heater mat adhered to an aluminum heat spreader plate that is thermally coupled to borosilicate glass (Fig. 3). The silicone heater mat has a built-in thermistor for temperature sensing and power wires connected to the Duet WiFi, which has integrated power control circuits and PID temperature control. The temperature of the heated bed plate is adjusted using the Panel Due touchscreen and can be maintained constant or changed throughout a stretching sequence through the use of specific g-code. A second thermistor can be added to the Duet WiFi and used to monitor the sample bath temperature.

### 2.3 The biaxial stretcher

**Fig. 4:**
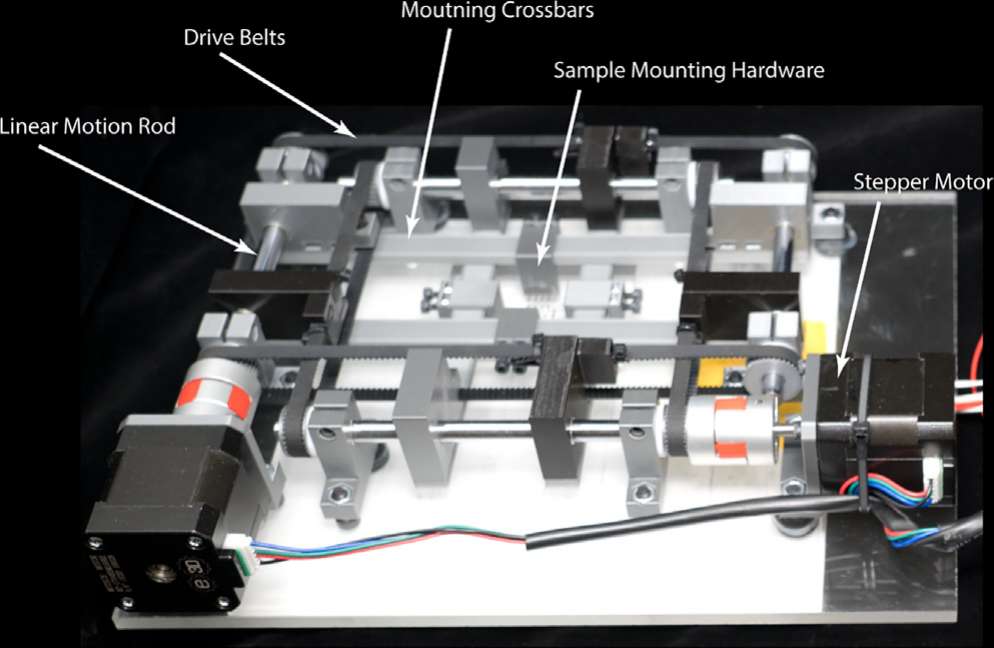
Assembled biaxial stretcher and sample mounting hardware.

The biaxial stretcher consists of a belt and pulley driven linear motion system with interchangeable sample mounting hardware assembled on a laser cut acrylic platform (Fig. 4). Two pairs of crossbars guided by linear motion rods are connected to opposite sides of a pulley mounted timing belt that stretches the sample on one axis symmetrically about the orthogonal axis. The linear motion rods serve a dual purpose, simultaneously guiding motion along one axis while acting as a driveshaft to impart motion along the orthogonal axis. Each axis is therefore run by a single stepper motor. This configuration allows for symmetrical biaxial motion in a compact form factor.

The biaxial stretcher uses interchangeable sample specific mounting hardware to attach a sample to the pairs of crossbars. These can consist of sample-penetrating rakes, clamping grips, or disposable adhesive based grips. These are 3D printed and easily swappable, with future designs for sample mounting hardware easily integrated into the system.

### 2.4 Key aspects of the hardware

• At a total cost of $580, the OBS is a fully customizable, open source, low cost alternative to commercial biaxial stretchers.
• The low-profile design of the OBS allows for live cell fluorescence imaging in combination with biaxially stretching in a variety of imaging platforms such as upright and inverted confocal microscopes, stereo microscopes, and overhead camera observation.
• The OBS integrated heated plate allows for temperature control required for live sample maintenance.
• Integration of the PanelDue 5i touchpad control provides quick and easy access to settings and motion control.
• The use of g-code enables customized stretching protocols in terms of magnitude, velocity, duration, and cycle frequency.

## 3 Design files

**Design Files Summary:** 

**Design File Name**	**Design File Type**	**Open Source License**	**Location of File**
AcrylicBuildPlate	CAD and PDF FIle	CC-BY 4.0	https://doi.org/10.5281/zenodo.3483849
SampleMediaTray	CAD and STL FIle	CC-BY 4.0	https://doi.org/10.5281/zenodo.3483849
CrossbeamAB	CAD and STL FIle	CC-BY 4.0	https://doi.org/10.5281/zenodo.3483849
CrossbeamCD	CAD and STL FIle	CC-BY 4.0	https://doi.org/10.5281/zenodo.3483849
CrossbeamEF	CAD and STL FIle	CC-BY 4.0	https://doi.org/10.5281/zenodo.3483849
CrossbeamGH	CAD and STL FIle	CC-BY 4.0	https://doi.org/10.5281/zenodo.3483849
BeltTensionerA Pt1	CAD and STL FIle	CC-BY 4.0	https://doi.org/10.5281/zenodo.3483849
BeltTensionerB Pt1	CAD and STL FIle	CC-BY 4.0	https://doi.org/10.5281/zenodo.3483849
BeltTensionerC Pt1	CAD and STL FIle	CC-BY 4.0	https://doi.org/10.5281/zenodo.3483849
BeltTensionerC Pt2	CAD and STL FIle	CC-BY 4.0	https://doi.org/10.5281/zenodo.3483849
BeltTensionerD Pt1	CAD and STL FIle	CC-BY 4.0	https://doi.org/10.5281/zenodo.3483849
BeltTensionerD Pt2	CAD and STL FIle	CC-BY 4.0	https://doi.org/10.5281/zenodo.3483849
BeltTensionerE Pt1	CAD and STL FIle	CC-BY 4.0	https://doi.org/10.5281/zenodo.3483849
BeltTensionerF Pt1	CAD and STL FIle	CC-BY 4.0	https://doi.org/10.5281/zenodo.3483849
BeltTensionerG Pt1	CAD and STL FIle	CC-BY 4.0	https://doi.org/10.5281/zenodo.3483849
BeltTensionerG Pt2	CAD and STL FIle	CC-BY 4.0	https://doi.org/10.5281/zenodo.3483849
BeltTensionerH Pt1	CAD and STL FIle	CC-BY 4.0	https://doi.org/10.5281/zenodo.3483849
BeltTensionerH Pt2	CAD and STL FIle	CC-BY 4.0	https://doi.org/10.5281/zenodo.3483849
RodSupport	CAD and STL FIle	CC-BY 4.0	https://doi.org/10.5281/zenodo.3483849
RodSupportRotatedFeet	CAD and STL File	CC-BY 4.0	https://doi.org/10.5281/zenodo.3483849
RodSupportRotatedFeetMirror	CAD and STL File	CC-BY 4.0	https://doi.org/10.5281/zenodo.3483849
MotorMount	CAD and STL FIle	CC-BY 4.0	https://doi.org/10.5281/zenodo.3483849
28ToothGT2Pulley	CAD and STL FIle	CC-BY 4.0	https://doi.org/10.5281/zenodo.3483849
32ToothGT2Pulley	CAD and STL FIle	CC-BY 4.0	https://doi.org/10.5281/zenodo.3483849
LongSampleMountingRakes	CAD and STL FIle	CC-BY 4.0	https://doi.org/10.5281/zenodo.3483849
ShortSampleMountingRakes	CAD and STL FIle	CC-BY 4.0	https://doi.org/10.5281/zenodo.3483849
SampleMountingShortClamp	CAD and STL FIle	CC-BY 4.0	https://doi.org/10.5281/zenodo.3483849
SampleMountingLongClamp	CAD and STL FIle	CC-BY 4.0	https://doi.org/10.5281/zenodo.3483849
SampleMountingClampTop	CAD and STL FIle	CC-BY 4.0	https://doi.org/10.5281/zenodo.3483849
RakeMountingJig	CAD and STL FIle	CC-BY 4.0	https://doi.org/10.5281/zenodo.3483849
RakeBendingJig	CAD and STL FIle	CC-BY 4.0	https://doi.org/10.5281/zenodo.3483849
DuetCaseBottom	CAD and STL FIle	CC-BY 4.0	https://doi.org/10.5281/zenodo.3483849
DuetCaseLid	CAD and STL FIle	CC-BY 4.0	https://doi.org/10.5281/zenodo.3483849
CaseAngles	CAD and STL FIle	CC-BY 4.0	https://doi.org/10.5281/zenodo.3483849
PanelDue5ICaseRear	CAD and STL FIle	CC-BY 4.0	https://doi.org/10.5281/zenodo.3483849
PanelDue5ICaseFront	CAD and STL FIle	CC-BY 4.0	https://doi.org/10.5281/zenodo.3483849
PowerSupplyMount	CAD and STL FIle	CC-BY 4.0	https://doi.org/10.5281/zenodo.3483849

### 3.1 Brief description of each part

**Duet WiFi and Panel Due Enclosure (**Fig. 20**):** The Duet Wifi is enclosed in a 3D printed box with ventilation grates (Adapted from www.thingiverse.com/thing:2799628 by LumberjackEngineering, and https://www.thingiverse.com/thing:2673206 by denos) with custom mounting brackets. The top of the enclosure has mounts for the PanelDue display enclosure. The stepper motor cables, heater bed plate cables, PanelDue cables, and input power are routed into this enclosure. The 12 V power supply is mounted to this enclosure using the PowerSupplyMountingPlate that fits through the ventilation grates and mounts to the power supply.

**Acrylic Build Platform (**Fig. 10**):** The acrylic build plate is laser cut to provide precise mounting locations for the stretching components and a windowed cutout for sample bath and use with an inverted microscope.

**Heated Bed Plate (**Fig. 3**):** A 200 × 200 mm silicone heater mat is adhered to a ⅛″ thick aluminum heat spreader. The heat spreader is coupled with silicone thermal tape to a ⅛″ borosilicate glass.

**Sample Bath:** This bath fits into the cutout in the acrylic build platform and can rest upon the heated bed plate. The bath allows the sample to remain submerged in the appropriate fluid maintained at the appropriate temperature.

**Stepper Motors (**Fig. 14**):** One stepper motor is needed for each of the orthogonal axes. These stepper motors stretch the samples along each axis using a belt and pulley transmission that turns their rotation into linear motion.

**MotorMounts (**Fig. 14**):** These stepper motor mounts are mounted at two specific precut locations on the acrylic platform. They support the stepper motors and prevent them from moving.

**Long (155 mm) and Short (140 mm) Linear Rods (**Fig. 10, Fig. 12**):** These linear rods, one pair for each orthogonal axis, simultaneously transfer the motion of the stepper motors to the pulley driven timing belts and allow for translation of the orthogonal axes along them. One each of the long and short rods is coupled to a stepper motor. These rods are supported by the acrylic platform mounted rod support housings.

**Stepper Motor Coupling (**Fig. 14**):** These couplers clamp to the stepper motor drive shaft and the linear rods to transfer the rotation of the stepper motors to the rods.

**RodSupports (**Fig. 9**):** These rod support housings are mounted at specific precut locations on the acrylic platform. Each housing has an 8 mm sleeve bearing through which the long and short linear rods run.

**28 and 32 tooth GT2 Pulleys (**Fig. 11, Fig. 13**):** These pulleys (28 tooth for the long linear rods and 32 tooth for the short linear rods) mate with the timing belts to transfer the rotation of the linear rods to the linear motion carriages.

**BeltTensionerC, D, G, H (**Fig. 10, Fig. 12, Fig. 16, Fig. 17**):** One pair of tensioning linear motion carriages is needed for each of the orthogonal axes. These carriages simultaneously tension the timing belts that drive the biaxial stretching motion and couple one each of the long and short crossbars to the belt.

**BeltTensionerA, B, E, F (**Fig. 10, Fig. 12, Fig. 16, Fig. 17**):** One pair of rider linear motion carriages is needed for each of the orthogonal axes. These carriages couple one each of the long and short crossbars to the belt.

**Crossbeams (**Fig. 10, Fig. 12**):** One pair each of long and short crossbars mount to the BeltTensioners on each of the orthogonal axes. These crossbars are where sample mounting hardware is affixed.

**Long and Short Sample Mounting Rakes and Jigs (**Fig. 18**):** These sample mounting rakes (2 each of the tall and short versions) are constructed by inserting prebent 23 gauge short bevel needles into the alignment holes within the mounting rakes. To align and permanently affix the needle rakes at various lengths, the needle alignment jig is used. The assembled mounting rakes are affixed to the pairs of long and short crossbars. The rakes are aligned at the center of the sample area. These rakes penetrate the sample and couple it to the motion of the long and short crossbars.

## 4 Bill of materials

**Table:** 

Designator	Component	Number of Units	Cost Per Unit [USD]	Total Cost [USD]	Source of Materials	Material Type
8 mm × 250 mm Linear Rods	B07JL1WK8B	2	11.39	22.78	Amazon	Steel
5 mm–8 mm Shaft Coupling	B06X9TVW64	2	12.39	24.78	Amazon	Aluminum
12 V Power Supply	B0109IMRPS	1	35.58	35.58	Amazon	Electronics
200 × 200 mm Silicone Heater	B011U6QCOA	1	38.99	38.99	Amazon	Silicone
20 mm × 0.15 mm × 25 m Thermal Silicone Double Sided Tape	B075FR45DV	1	11.99	11.99	Amazon	Silicone
Sleeve Bearings	6679K13	16	2.77	44.32	McMaster	Steel/PTFE
M3 Thin Nut	90695A033	36	0.033	1.188	McMaster	Steel
M5 Thin Nut	90695A037	20	0.0298	0.596	McMaster	Steel
Borosilicate Glass 8″ × 8″ × ⅛″	8476K18	1	25.00	25.00	McMaster	Glass
6061 Aluminum Sheet 8″ × 8″ × ⅛″	89015K239	1	13.86	13.86	McMaster	Aluminum
12″ × 12″ × ¼″ Acrylic Sheet	4615T37	1	12.43	12.43	McMaster	Acrylic
M3 x 0.5 3 mm Long Set Screw	91390A097	8	0.044	0.352	McMaster	Steel
PTFE Washer	95630A670	8	0.485	3.88	McMaster	PTFE
M5 Low- Profile socket Head Bolt	93070A125	20	0.2262	4.52	McMaster	Steel
Rubber Grommet	9600K32	8	0.078	0.624	McMaster	Rubber
Black PLA	MY6CYEZM	1	19.99	19.99	Matter Hackers	PLA
Duet 2 WiFi	Duet 2 Wifi, Standard, No Addons	1	169.99	169.99	Filastruder	Electronics
Panel Due I5	PanelDue 5I with 4 pin, 1 m cable	1	89.99	89.99	Filastruder	Electronics
.9 Degree Stepper Motor	High Torque Axis Motor	2	24.99	49.98	Filastruder	Electronics
Stepper Motor Cable	Stepper Motor Cable	2	2.99	5.98	Filastruder	Electronics
M3 x 0.5 10 mm Long Socket Head Bolt	91290A115	20	0.0736	1.472	McMaster	Steel
M3 x 0.5 25 mm Long Socket Head Bolt	91290A125	4	0.1289	0.5156	McMaster	Steel
M3 x 0.5 16 mm Long Socket Head Bolt	91290A120	4	0.0887	0.7096	McMaster	Steel
M3 x 0.5 14 mm Long Socket Head Bolt	91290A119	8	0.111	0.888	McMaster	Steel

## 5 Build instructions

The majority of the OBS is 3D printed from PLA filament. All STL files have been prepared and saved in their intended printing orientation. During the printing process no supports are required. All parts were printed with 60% infill and 2 perimeters with a layer height of 0.250 mm. Some print settings may vary depending upon the printer used (Table Design Files Summary).

### 5.1 AB crossbeam assembly

**Fig. 5:**
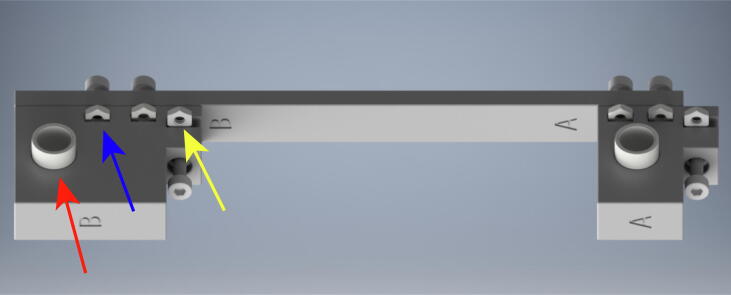
Render of AB crossbeam assembly.

Begin assembly of the OBS by constructing the crossbeams, the first crossbeam is the AB crossbeam (Fig. 5). For this assembly you need:

(1x) BeltTensionerA Pt1
(1x) BeltTensionerB Pt1
(1x) CrossbeamAB
(2x) High-Load Dry-Running Bearing with Steel Shell
(6x) 10 mm M3x.5 mm Socket Head Bolt
(6x) Metric Medium-Strength Steel Thin Hex Nuts

Press fit one bearing each into BeltTensionerA Pt1 and BeltTensionerB Pt1 (red arrow). Insert two thin hex nuts into the slots (blue arrow) on each and tighten to matching side of CrossbeamAB using the 10 mm M3 bolts. Finally insert one hex nut into the recess of each BeltTensioner’s clamp (yellow arrow) and thread a 10 mm M3 bolt.

### 5.2 CD crossbeam assembly

**Fig. 6:**
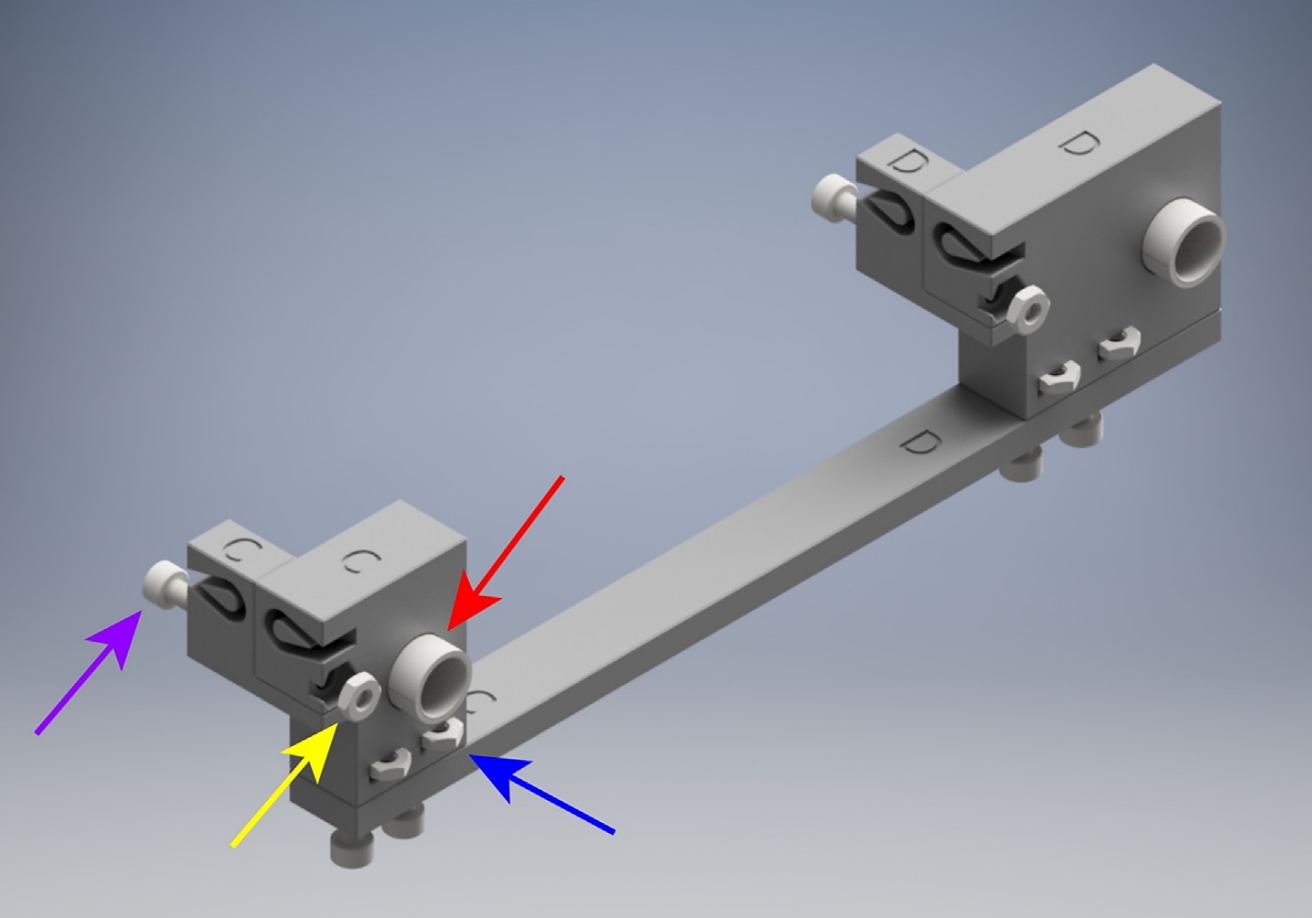
Render of CD crossbeam assembly.

Assemble the second crossbeam, the CD crossbeam (Fig. 6). For this assembly you need:

(1x) BeltTensionerC Pt1
(1x) BeltTensionerC Pt2
(1x) BeltTensionerD Pt1
(1x) BeltTensionerD Pt2
(1x) CrossbeamCD
(2x) High-Load Dry-Running Bearing with Steel Shell
(4x) 10 mm M3x.5 mm Socket Head Bolt
(6x) Metric Medium-Strength Steel Thin Hex Nuts
(2x) 25 mm M3x.5 mm Socket Head Bolt

Press fit one bearing each into BeltTensionerC Pt1 and BeltTensionerD Pt1 (red arrow). Insert two thin hex nuts into the slots (blue arrow) on each and tighten to matching side of CrossbeamCD using 10 mm M3 bolts. Insert one hex nut into the recess of each BeltTensioner’s clamp (yellow arrow). Finally thread a 25 mm M3 bolt (purple arrow) through each BeltTensionerC Pt2 and BeltTensionerD Pt2 to attach to the corresponding Pt1 components.

### 5.3 EF crossbeam assembly

**Fig. 7:**
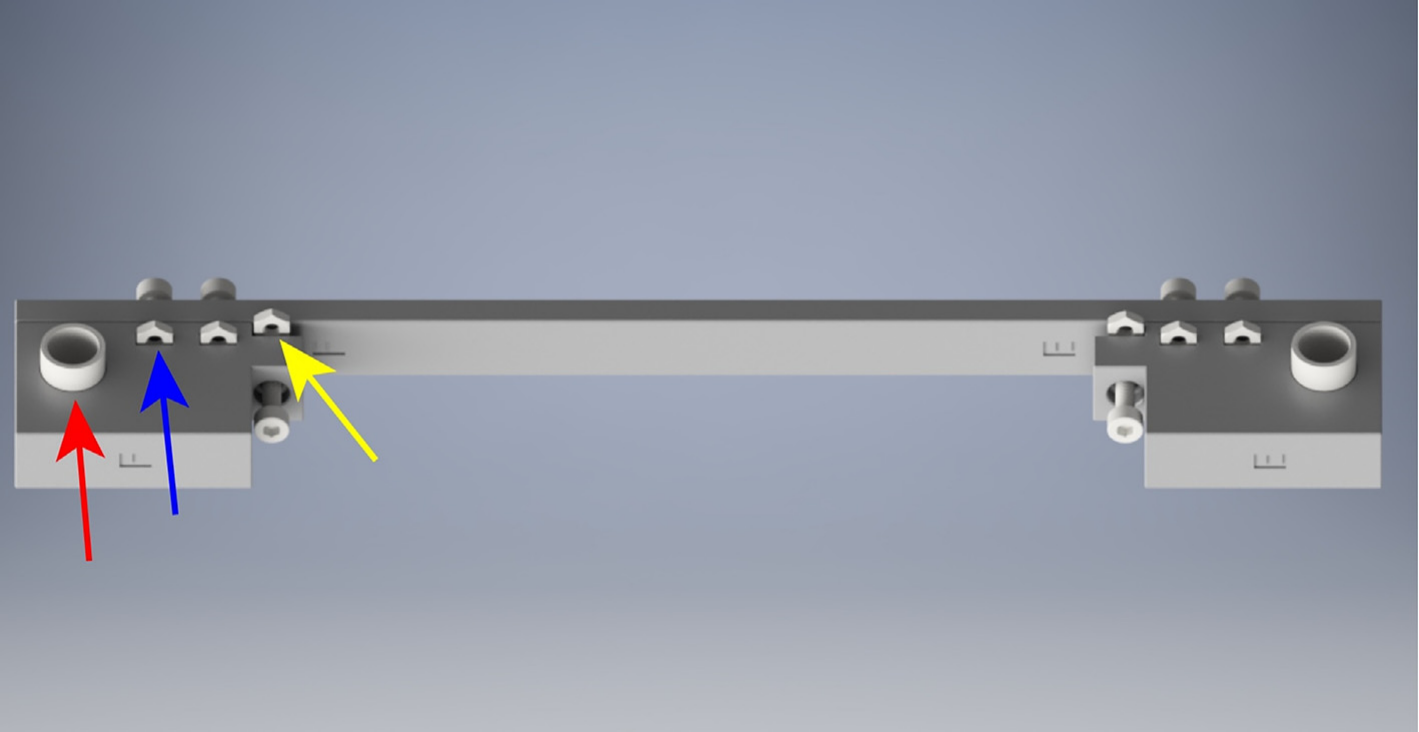
Render of EF crossbeam assembly.

Assemble the third crossbeam, the EF crossbeam (Fig. 7). For this assembly you need:

(1x) BeltTensionerE Pt1
(1x) BeltTensionerF Pt1
(1x) CrossbeamEF
(2x) High-Load Dry-Running Bearing with Steel Shell
(6x) 10 mm M3x.5 mm Socket Head Bolt
(6x) Metric Medium-Strength Steel Thin Hex Nuts

Press fit one bearing each into BeltTensionerE Pt1 and BeltTensionerF Pt1 (red arrow). Insert two thin hex nuts into the slots (blue arrow) on each and tighten to matching side of CrossbeamEF using the 10 mm M3 bolts. Finally insert one hex nut into the recess of each BeltTensioner’s clamp (yellow arrow) and thread a 10 mm M3 bolt.

### 5.4 GH crossbeam assembly

**Fig. 8:**
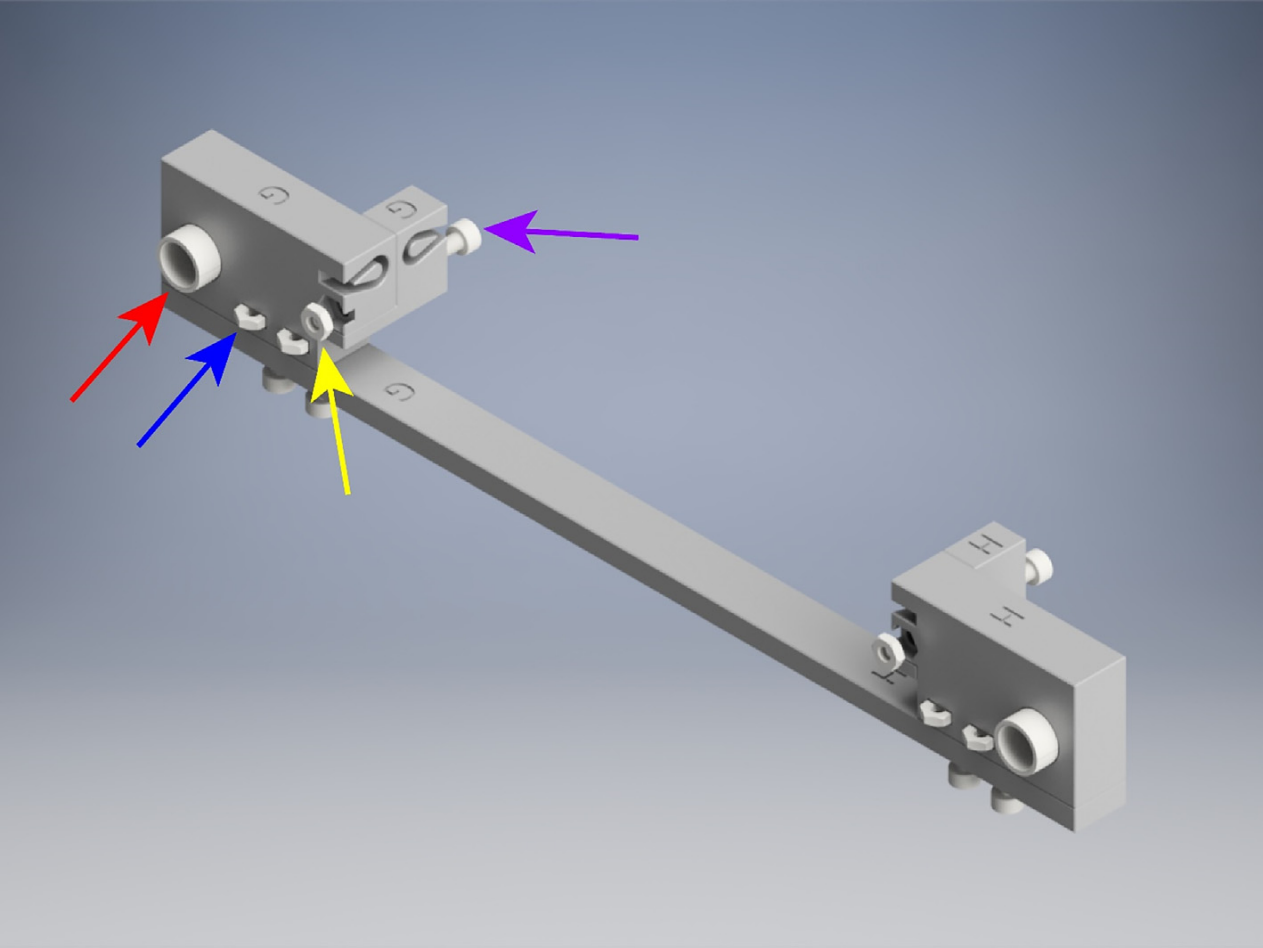
Render of GH crossbeam assembly.

Assemble the fourth crossbeam, the GH crossbeam (Fig. 8). For this assembly you need:

(1x) BeltTensionerG Pt1
(1x) BeltTensionerG Pt2
(1x) BeltTensionerH Pt1
(1x) BeltTensionerH Pt1
(1x) CrossbeamGH
(2x) High-Load Dry-Running Bearing with Steel Shell
(4x) 10 mm M3x.5 mm Socket Head Bolt
(6x) M3 Metric Medium-Strength Steel Thin Hex Nuts
(2x) 25 mm M3x.5 mm Socket Head Bolt

Press fit one bearing each into BeltTensionerG Pt1 and BeltTensionerH Pt1 (red arrow). Insert two thin hex nuts into the slots (blue arrow) on each and tighten to matching side of CrossbeamGH using 10 mm M3 bolts. Insert one hex nut into the recess of each BeltTensioner’s clamp (yellow arrow). Finally thread a 25 mm M3 bolt (purple arrow) through each BeltTensionerG Pt2 and BeltTensionerH Pt2 to attach to the corresponding Pt1 components.

### 5.5 RodSupport assembly

**Fig. 9:**
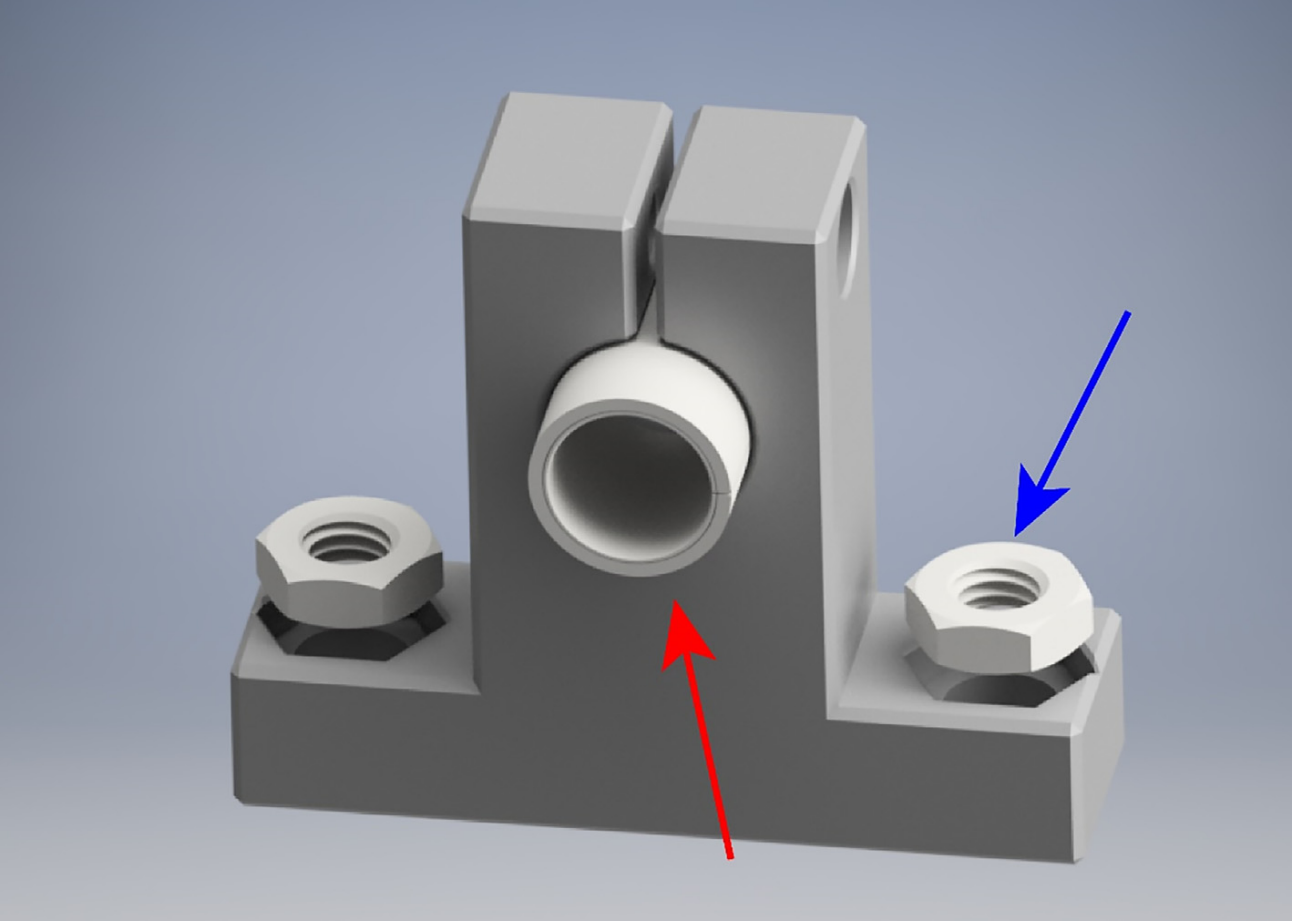
Render of RodSupport assembly.

Assemble the 8 RodSupports (Fig. 9). You need:

(6x) RodSupport
(1x) RodSupport Rotated Feet
(1x) RodSupport Rotated Feet Mirror
(16x) M5 Metric Medium-Strength Steel Thin Hex Nuts
(8x) High-Load Dry-Running Bearing with Steel Shell

Press fit one bearing (red arrow) into each of the RodSupport pieces. Next Fit two M5 thin hex nuts into each RodSupport Piece (blue arrow).

### 5.6 Short axis mounting step

**Fig. 10:**
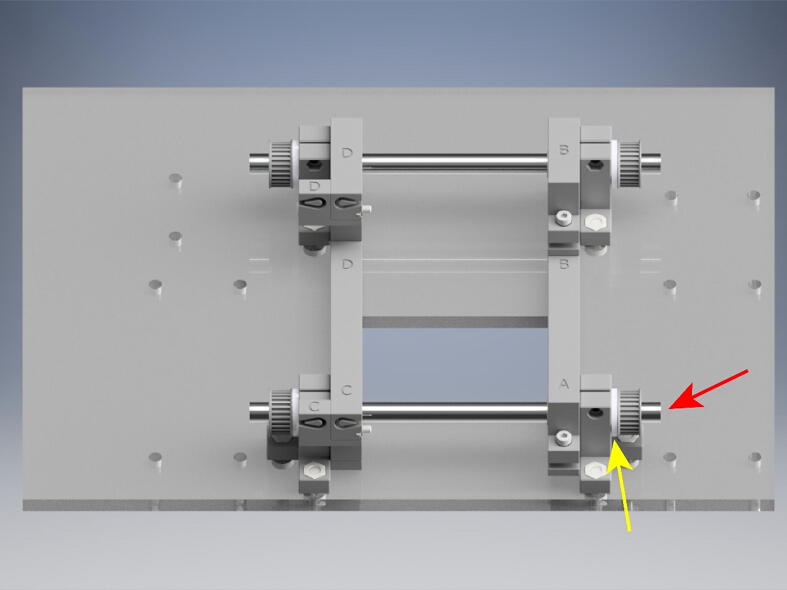
Render of short axis mounting step.

Begin to mount the OBS to the acrylic build platform (Fig. 10). For this you will need:

(1x) AB Crossbeam Assembly
(1x) CD Crossbeam Assembly
(2x) RodSupport
(1x) RodSupport Rotated Feet
(1x) RodSupport Rotated Feet Mirror
(4x) 28 Tooth GT2 Pulley
(4x) PTFE Washer
(8x) 16 mm M5 Low-Profile Socket Head Bolt
(2x) 155 mm long 8 mm diameter linear rod
(4x) M3 Metric Medium-Strength Steel Thin Hex Nuts
(4x) 3 mm M3x.5 mm Cup Point Set Screw

First, insert the 155 mm long 8 mm diameter linear rods (red arrow) into either side of the AB and CD Crossbeam assemblies. Center the assemblies on the linear rods. Next, on the B and D side add 1 RodSupport assembly to either side of the Crossbeam assemblies. Next, on the C side add the RodSupport Rotated Feet assembly and on the A side add the RodSupport Rotated Feet Mirror assembly. Align these RodSupport assemblies with the corresponding holes in the acrylic build platform. Thread 16 mm M5 Low-Profile socket head bolts through each hole and fasten the assemblies to the acrylic build platform. Next place one PTFE washer (yellow arrow) on the outer sides of the RodSupport Assemblies.

**Fig. 11:**
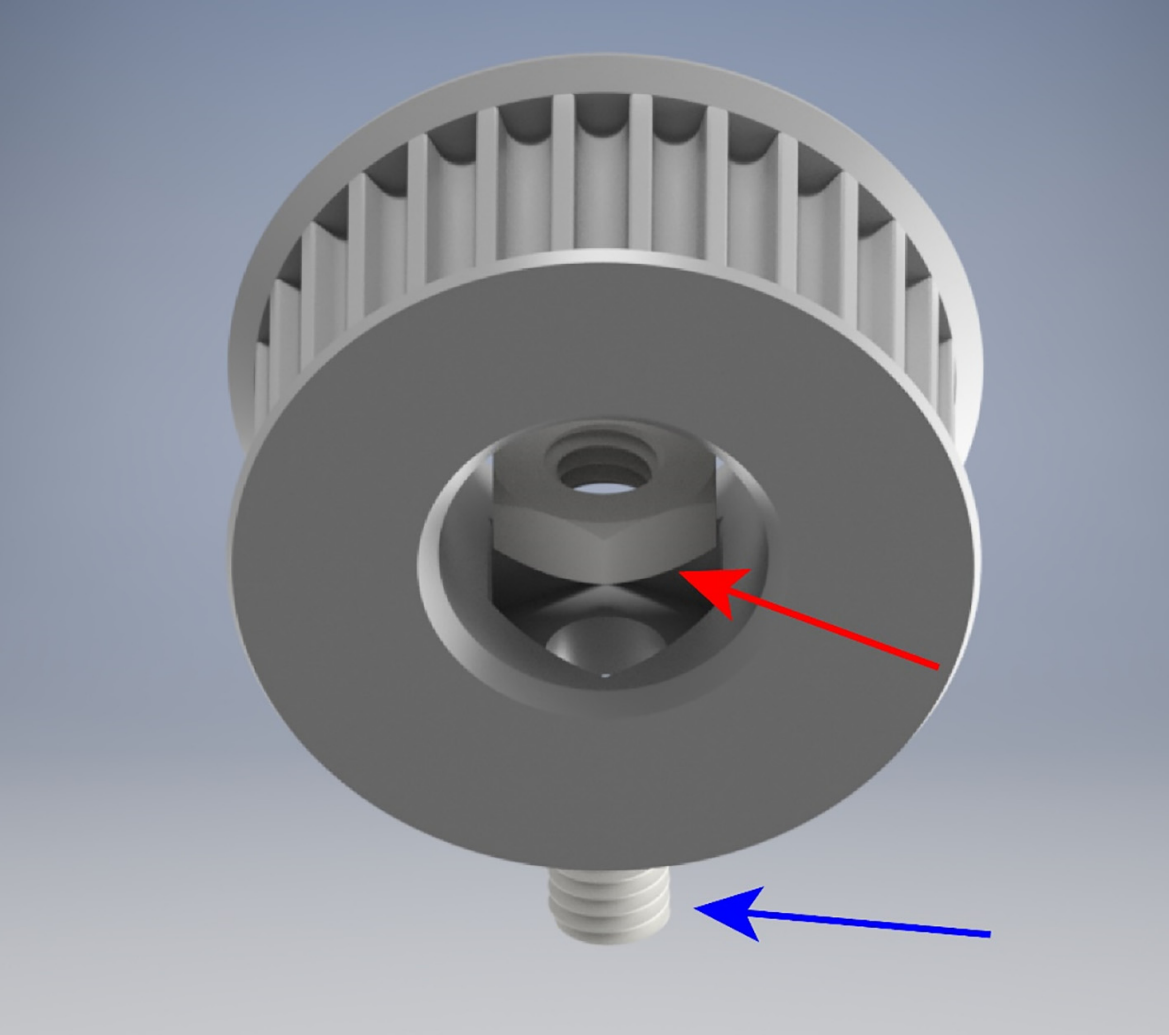
Render of 28 tooth GT2 pulley assembly.

Insert one M3 thin hex nut into the backside of the pulley (Fig. 11, red arrow). While sliding the pulley onto the ends of the 155 mm long 8 mm diameter linear rods this thin hex nut can be retained by threading a 10 mm long M3x.5 mm socket head bolt into the nut until it just catches. Once the pulley and nut are on the linear rod the socket head bolt can be replaced with a 3 mm M3x.5 mm cup point set screw (blue arrow).

Adjust the linear rods until the lengths protruding from either side of the RodSupports are equivalent. Affix the pulleys to the rod by tightening the set screws in each. Ensure that the pulleys are pressed up against the PTFE washers and that there is no motion of the linear rod along its axis.

### 5.7 Long axis mounting step

**Fig. 12:**
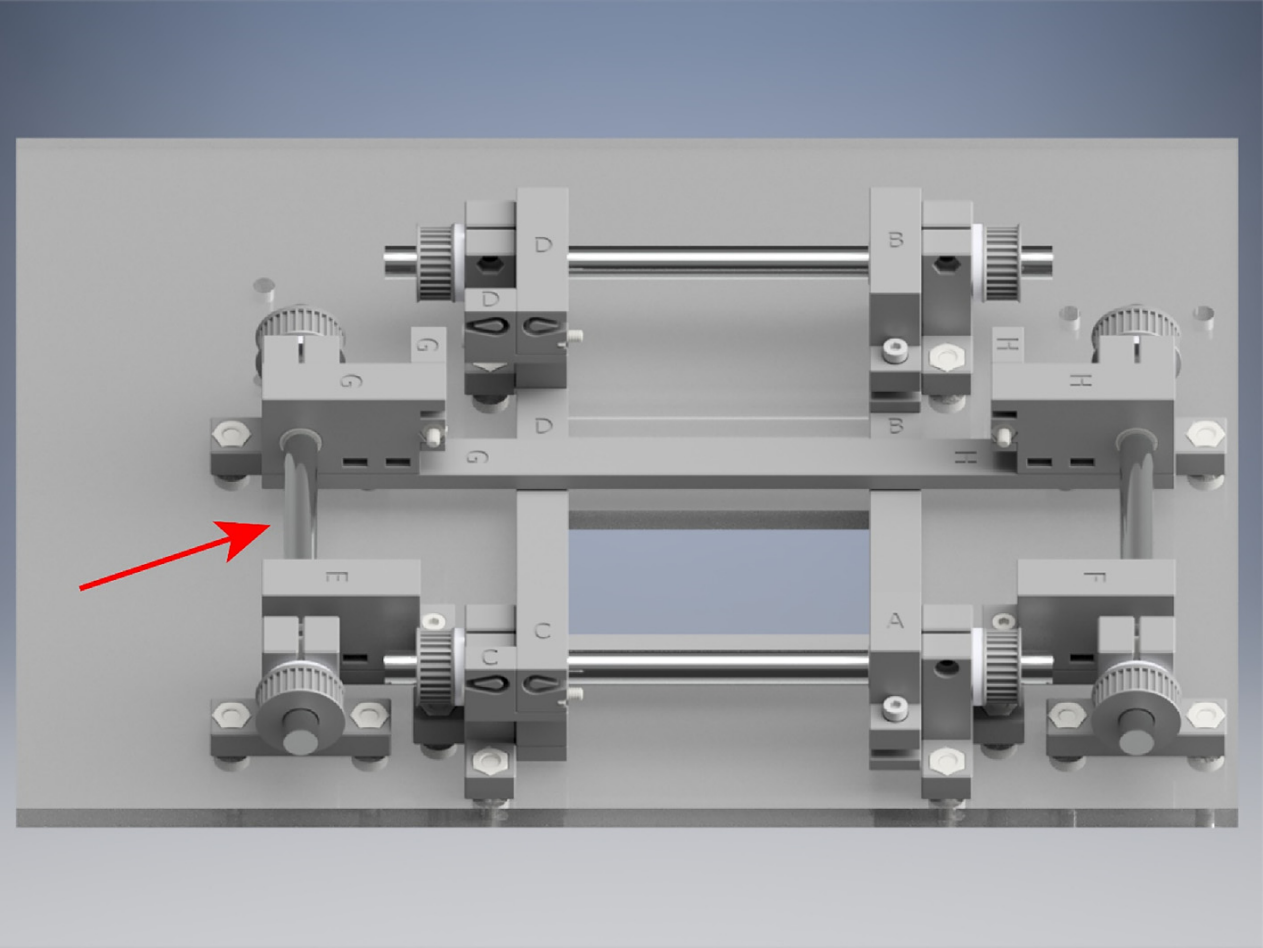
Render of long axis mounting step.

Continue to mount the OBS to the acrylic build platform (Fig. 12). For this you will need:

(1x) EF Crossbeam Assembly
(1x) GH Crossbeam Assembly
(4x) RodSupport
(4x) 32 Tooth GT2 Pulley
(4x) PTFE Washer
(8x) 16 mm M5 Low-Profile Socket Head Bolt
(2x) 140 mm long 8 mm diameter linear rod
(4x) M3 Metric Medium-Strength Steel Thin Hex Nuts
(4x) 3 mm M3x.5 mm Cup Point Set Screw

First, insert the 140 mm long 8 mm diameter linear rods (red arrow) into either side of the EF and GH Crossbeam assemblies. Center the assemblies on the linear rods. Next, on both sides of the assemblies add 1 RodSupport assembly to either side of the Crossbeam assemblies. Align these RodSupport assemblies with the corresponding holes in the acrylic build platform. Thread 16 mm M5 Low-Profile socket head bolts through each hole and fasten the assemblies to the acrylic build platform. Next place one PTFE washer on the outer sides of the RodSupport Assemblies.

**Fig. 13:**
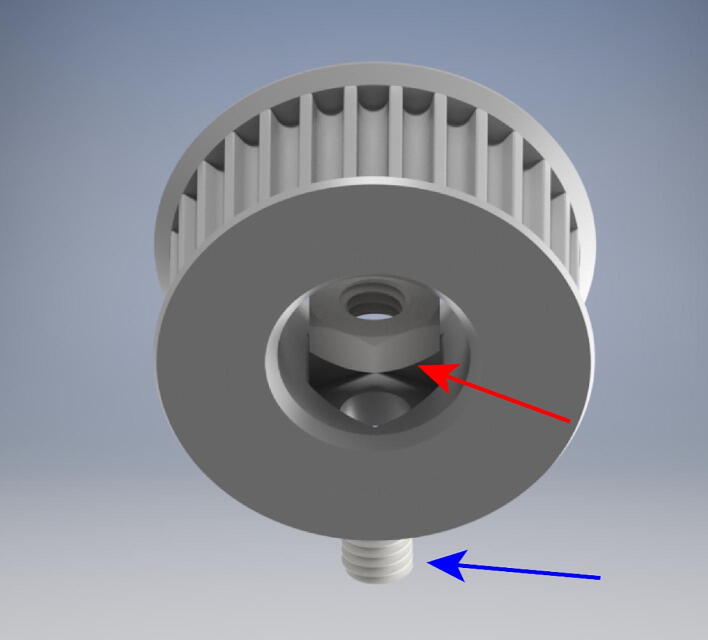
Render of 32 tooth GT2 pulley.

Assemble the 32 tooth GT2 Pulleys:

Insert one M3 thin hex nut into the backside of the pulley (Fig. 13, red arrow). While sliding the pulley onto the ends of the 140 mm long 8 mm diameter linear rods this thin hex nut can be retained by threading a 10 mm long M3x.5 mm socket head bolt into the nut until it just catches. Once the pulley and nut are on the linear rod the socket head bolt can be replaced with a 3 mm M3x.5 mm cup point set screw (blue arrow).

Adjust the linear rods until the lengths protruding from either side of the RodSupports are equivalent. Affix the pulleys to the rod by tightening the set screws in each. Ensure that the pulleys are pressed up against the PTFE washers and that there is no motion of the linear rod along its axis.

### 5.8 Motor mount assembly

**Fig. 14:**
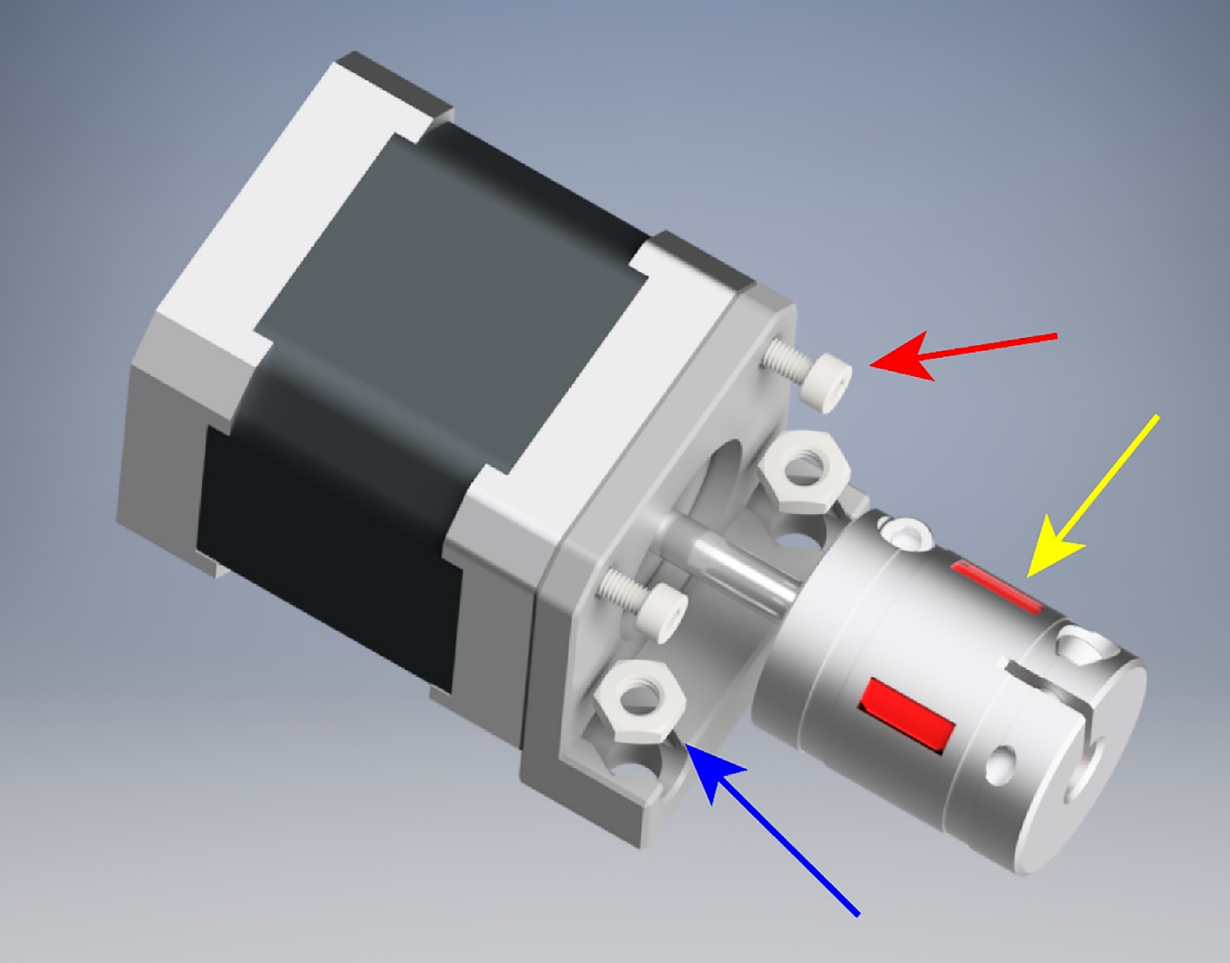
Render of motor and coupler assembly.

Assemble the two motor assemblies (Fig. 14), you will need:

(2x) 0.9-degree NEMA 17 Stepper motor
(4x) 10 mm M3x.5 mm Socket Head Bolts
(1x) MotorMount
(4x) M5 Metric Medium-Strength Steel Thin Hex Nuts
(2x) 5 mm to 8 mm shaft coupling

For each assembly mount the stepper motor to the MotorMount using 2 10 mm long M3x.5 mm socket head bolts (red arrow). Next, insert two M5 thin hex nuts into their recesses (blue arrow). Finally, clamp the 5 mm side of the shaft coupling to the stepper motor shaft (yellow arrow).

### 5.9 Motor mounting step

**Fig. 15:**
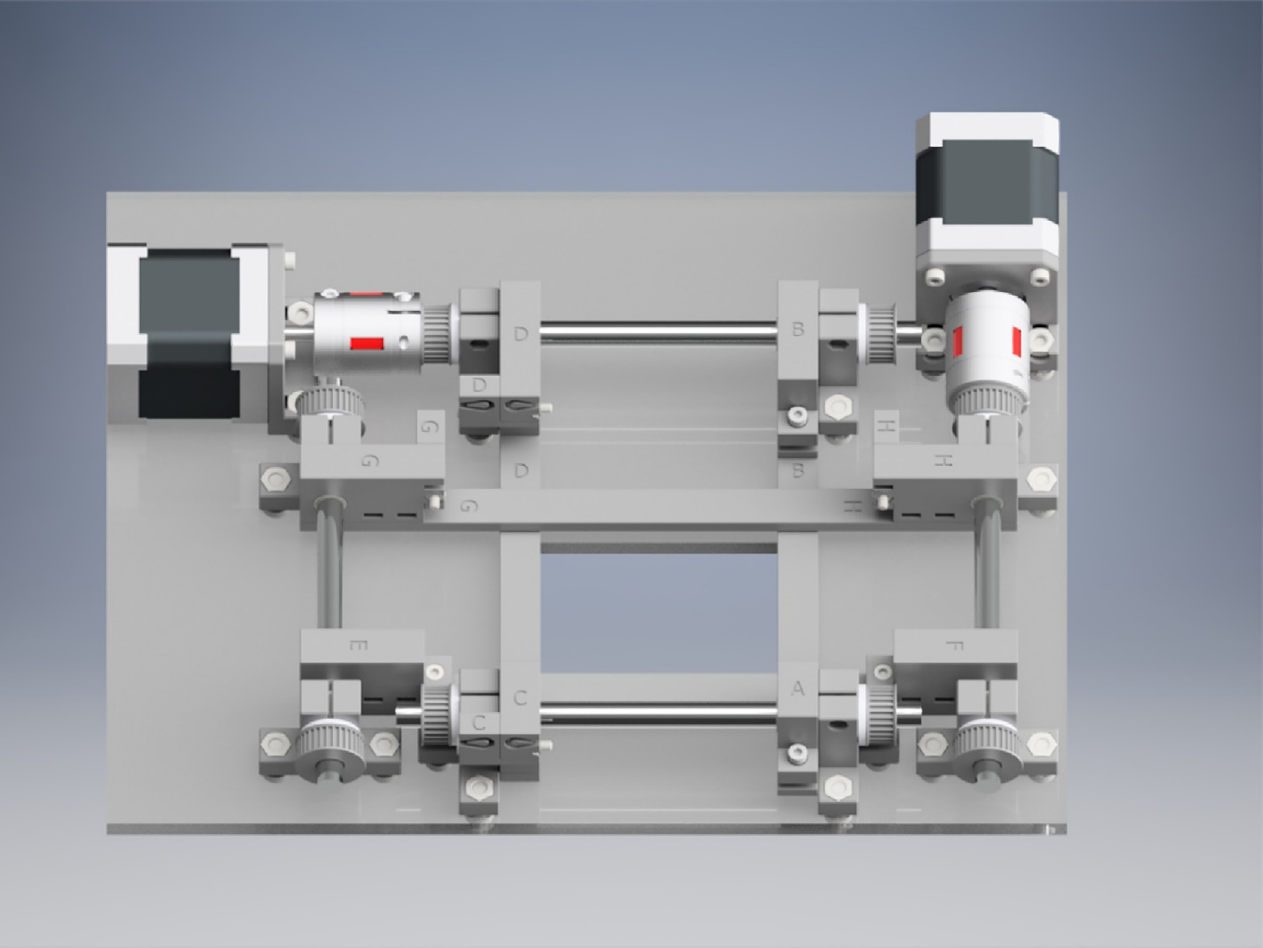
Render of motor mounting step.

Continue to mount the OBS to the acrylic build platform (Fig. 15). For this you will need:

(2x) Motor Assemblies
(4x) 16 mm M5 Low-Profile Socket Head Bolt

Loosen the clamping bolts on the shaft couplings to allow them to slide along the motor shaft. For each motor assembly slide the shaft coupling onto the corresponding 8 mm linear rod. Align the holes in the motor mounts with the associated holes in the acrylic build platform. Thread 16 mm M5 Low-Profile socket head bolts through each hole and fasten the assemblies to the acrylic build platform. Adjust the shaft couplings so that they capture a sufficient portion of the 5 mm motor shafts and the 8 mm linear rods. Next, tighten the shaft coupling clamping bolts.

### 5.10 Drive belt fitting

**Fig. 16:**
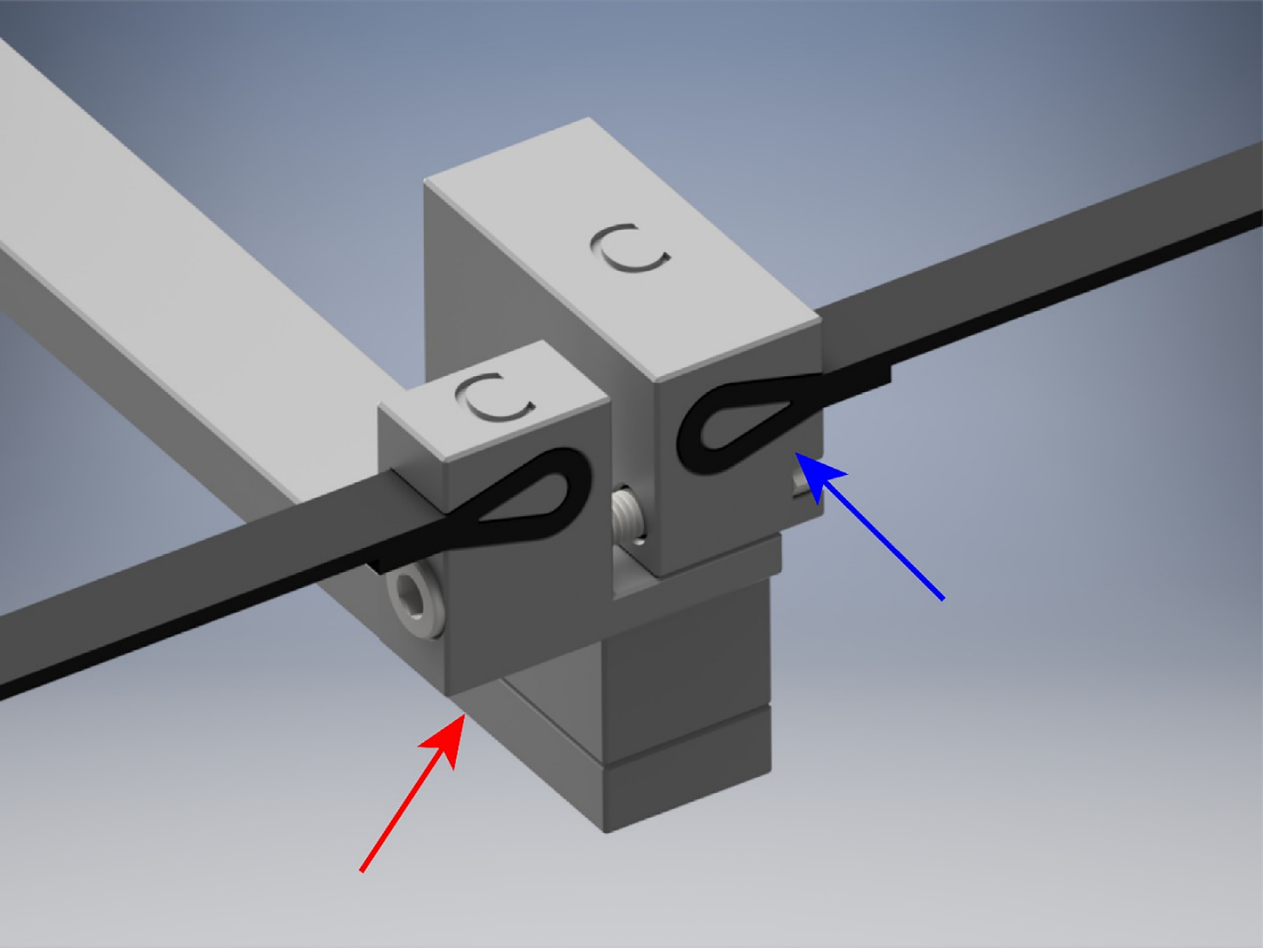
Render of GT2 belting into tensioning elements.

Continue to assemble the OBS (Fig. 16). For this you will need:

(2x) Long 490 mm GT2 Belts
(2x) Short 360 mm GT2 Belts
(4x) Small zipties

From the long roll of GT2 belt, cut belts for each component of the OBS. Loosen the tensioning elements on parts C, D, G, and H (red arrow) and measure the short and long belts such that there will be 1 cm of extra belt on either side of the belt recesses (blue arrow). Insert the belt into the belt loop recesses (blue arrow). The belt teeth will interlock making a secure connection.

### 5.11 Belt attachment and tensioning

**Fig. 17:**
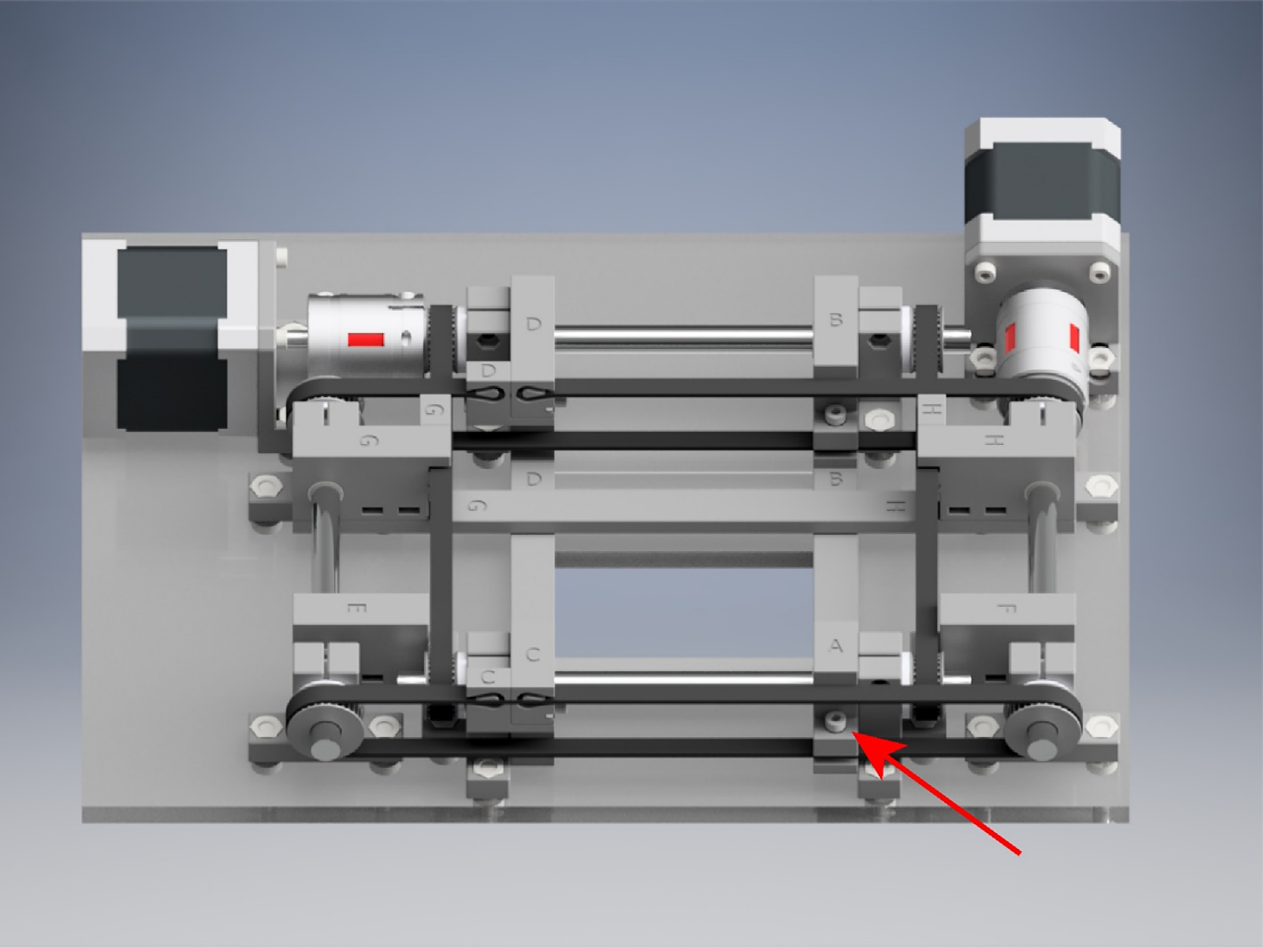
Render of belt attachment and tensioning.

After cutting the two short and long GT2 belts, wrap them around the pulleys, starting with the two shorter belts (Fig. 17). Feed the ends into the belt recesses, trying to keep the belt taut. Zip tie the excess belt as close to the end loops as possible. Trim the belt close to the zip tie. Repeat with the longer belts, making sure to loop around the outside of the small belt loops. After the belts have been secured, tighten the 25 mm M3x.5 mm screws to increase tension in the belts. Tension until taut. But do not over tighten to avoid plastic distortion. Expand the OBS to its maximum stretch, as in the above picture. On the A, B, E, and F crossbeam elements tighten the 10 mm M3x.5 mm socket head bolt (red arrow) to couple the crossbeam to the belt.

### 5.12 Rake jig assembly for sample holding

**Fig. 18:**
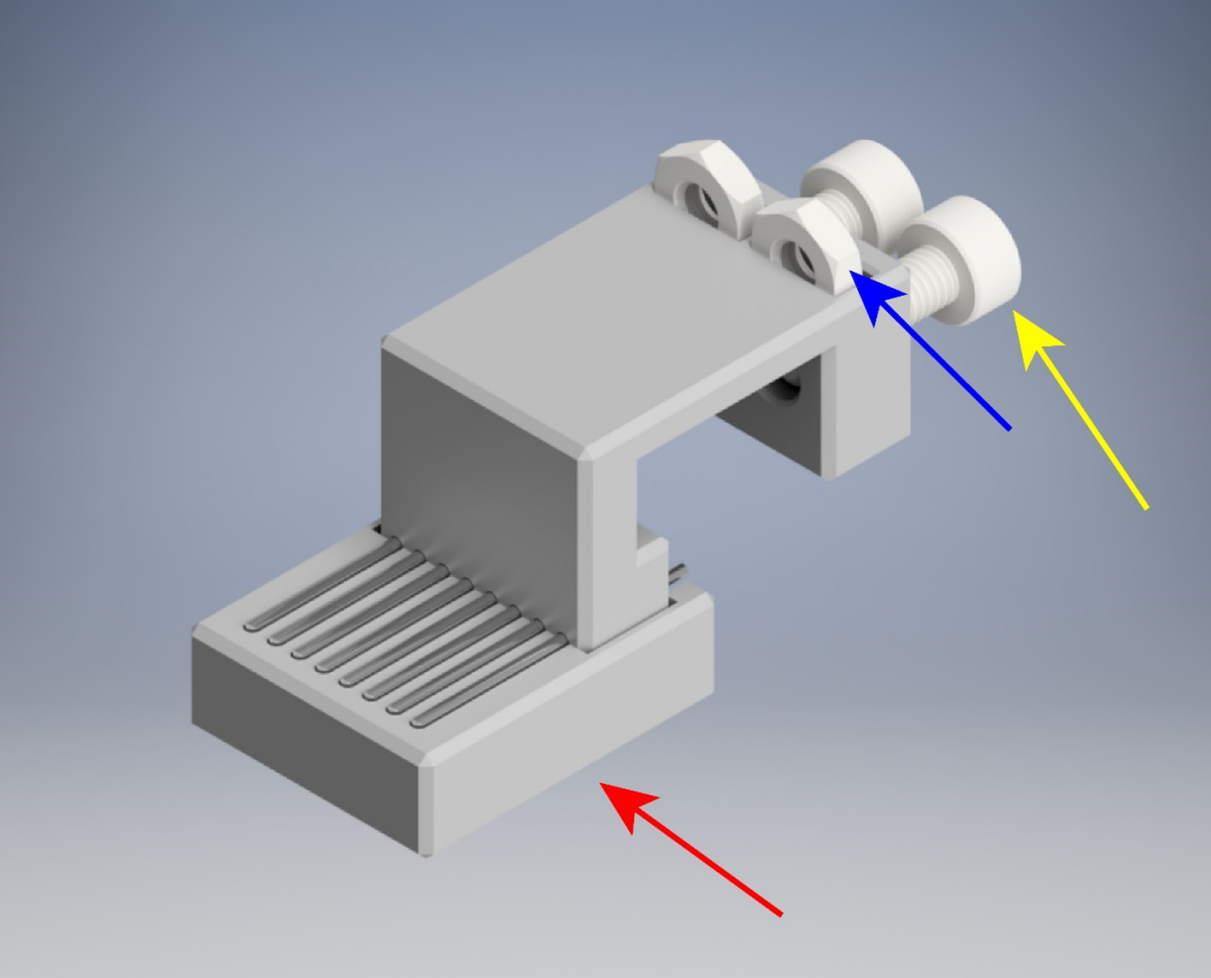
Render of rake assembly jig.

Next, assemble the sample mounting rakes (Fig. 18), for this you will need:

(32x) 23 g needles
(1x) RakeBendingJig
(2x) LongSampleMountingRake
(2x) ShortSampleMountingRake
(4x) RakeMountingJig
(8x) M3 Metric Medium-Strength Steel Thin Hex Nuts
(8x) 10 mm M3x.5 mm Socket Head Bolts

Bend the 32 needles using the rake bending jig. Next, insert 8 bent rakes into each of the long and short sample mounting rake parts. Apply superglue to the rear of the needles and align with the associated rake mounting jigs (red arrow). Clamp and let sit to cure, according to your glue. To finish assembly slot 2 M3 thin nuts into each sample mounting rake (blue arrow), and thread one 10 mm M3 socket head bolt (yellow arrow) into each of those.

### 5.13 Sample rake mounting to crossbeams

**Fig. 19:**
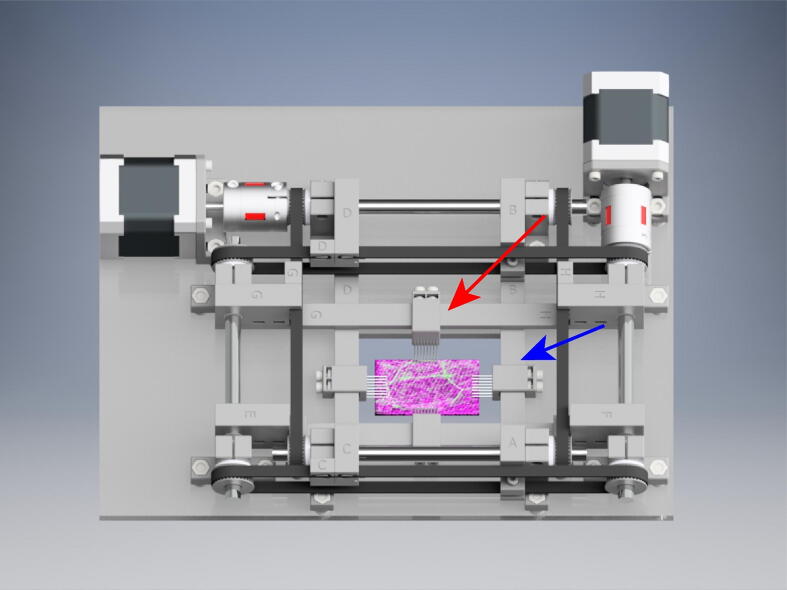
Render of sample rake mounting to crossbeams.

To finish assembly of the OBS (Fig. 19), you will need:

(2x) Long Sample Mounting Rake
(2x) Short Sample Mounting Rake

Mount the 2 long sample mounting rakes (red arrow) to the opposing GH and EF crossbeams by gently tightening the 10 mm M3 socket head bolts in each rake. Mount the 2 short sample mounting rakes (blue arrow) to the opposing AB and CD crossbeams by gently tightening the 10 mm M3 socket head bolts in each rake. Mount sample by placing under the sample rakes and pressing upwards into the needle array. Alternative means of sample mounting and grasping can be used and designed to fit onto the crossbeams.

### 5.14 Duet WiFi and PanelDue 5i assembly

**Fig. 20:**
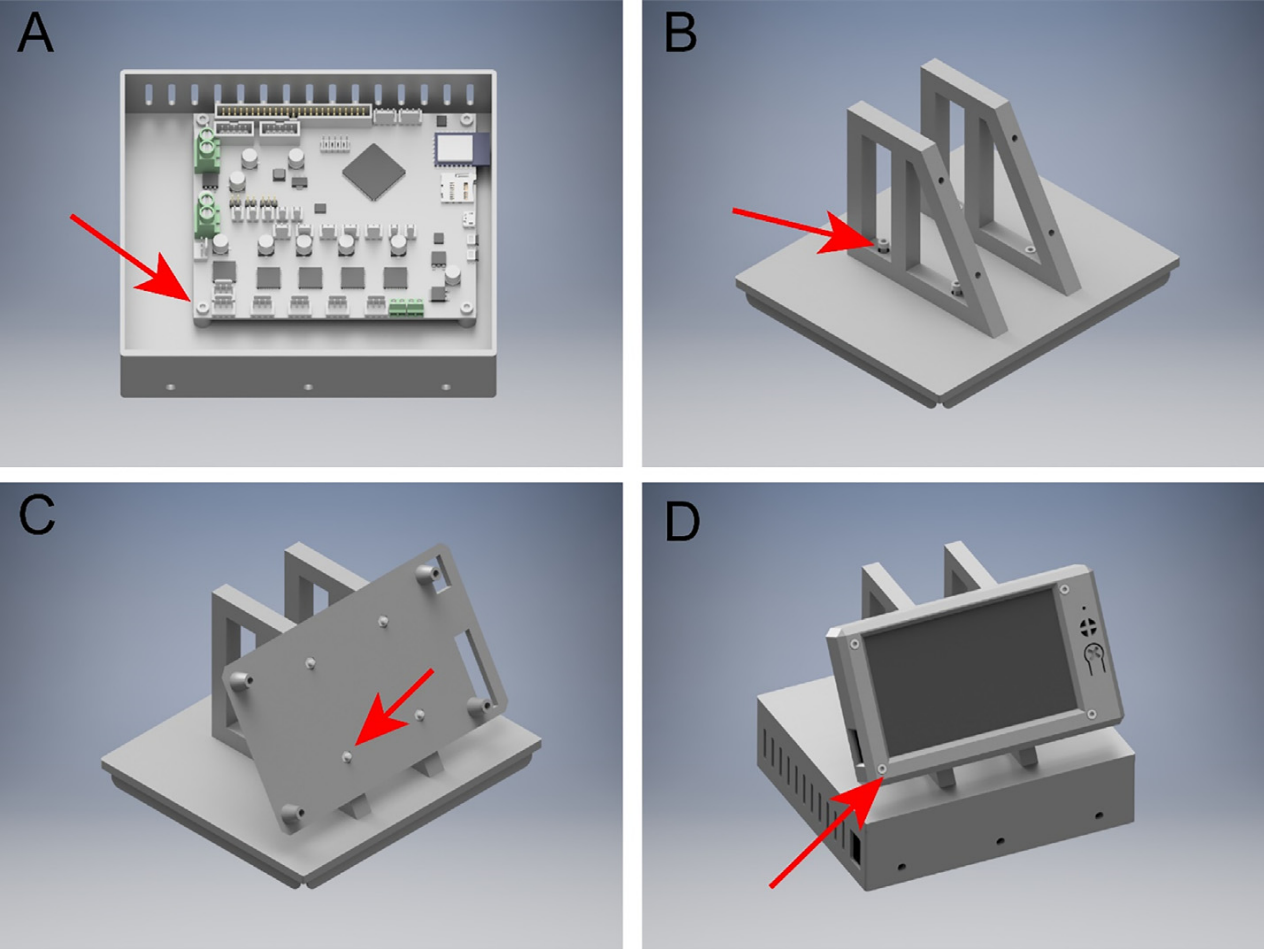
Render of Duet WiFi and PanelDue 5i assembly.

To assemble the Duet WiFi Enclosure with PanelDue 5I (Fig. 20), you will need:

(1x) Duet Wifi
(1x) PanelDue 5I
(1x) DuetCaseBottom
(1x) DuetCaseLid
(2x) CaseAngles
(1x) PanelDue5ICaseRear
(1x) PanelDue5ICaseFront
(8x) 10 mm M3x.5 mm Socket Head Bolts
(8x) 14 mm M3x.5 mm Socket Head Bolts
(8x) M3 Metric Medium-Strength Steel Thin Hex Nuts
(1x) PowerSupplyMountingPlate

Bolt the Duet Wifi to the DuetCaseBottom with 4 10 mm M3 socket head bolts (Fig. 20A). Next, attach the CaseAngles to the DuetCaseLid using 4 14 mm M3 socket head bolts and 4 M3 thin nuts (Fig. 20B). Attach the PanelDue5ICaseRear to the CaseAngles using 4 14 mm M3 socket head bolts and 4 M3 thin nuts (Fig. 20C). Finally, attach the PanelDue5ICaseFront and PanelDue 5I by threading 4 10 mm M3 socket head bolts through the PanelDue 5I into the standoffs in the PanelDue5ICaseRear (Fig. 20D). Place the DuetCaseLid on the DuetCaseBottom. Mount the power supply on the back of the DuetCaseBottom using the PowerSupplyMounntingPlate. For instructions setting up the DuetWifi and PanelDue 5I go to https://duet3d.dozuki.com/

## 6 Operating instructions

**Fig. 21:**
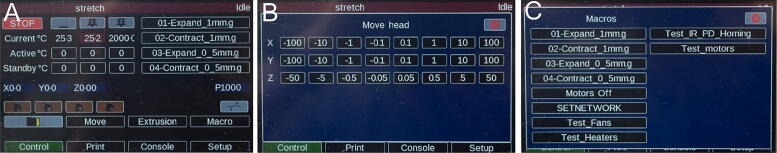
Images of PanelDue control screens. (A) Main screen (B) Move screen (C) Macro Screen.

To operate the OBS first power it on. The PanelDue 5i comes preloaded with a graphical user interface to control the Duet WiFi. Select the [Active °C] button on the main screen of the PanelDue 5i to begin warming the heated bed plate to the temperature required for the experiment (Fig. 21A). Mount the sample on the sample rakes by penetrating the sample with the needle tips. Use the move button on the PanelDue to access the screen for movement of the axes individually (Fig. 21B). Move each axis until the slack in the sample is taken up. To stretch and contract the sample you can use the macros we have included as downloadable files or generate your own using the MATLAB script we have provided. These macros can be accessed on the main screen of the PanelDue, as well as the macro screen (Fig. 21C).

## 7 Validation and characterization

**Fig. 22:**
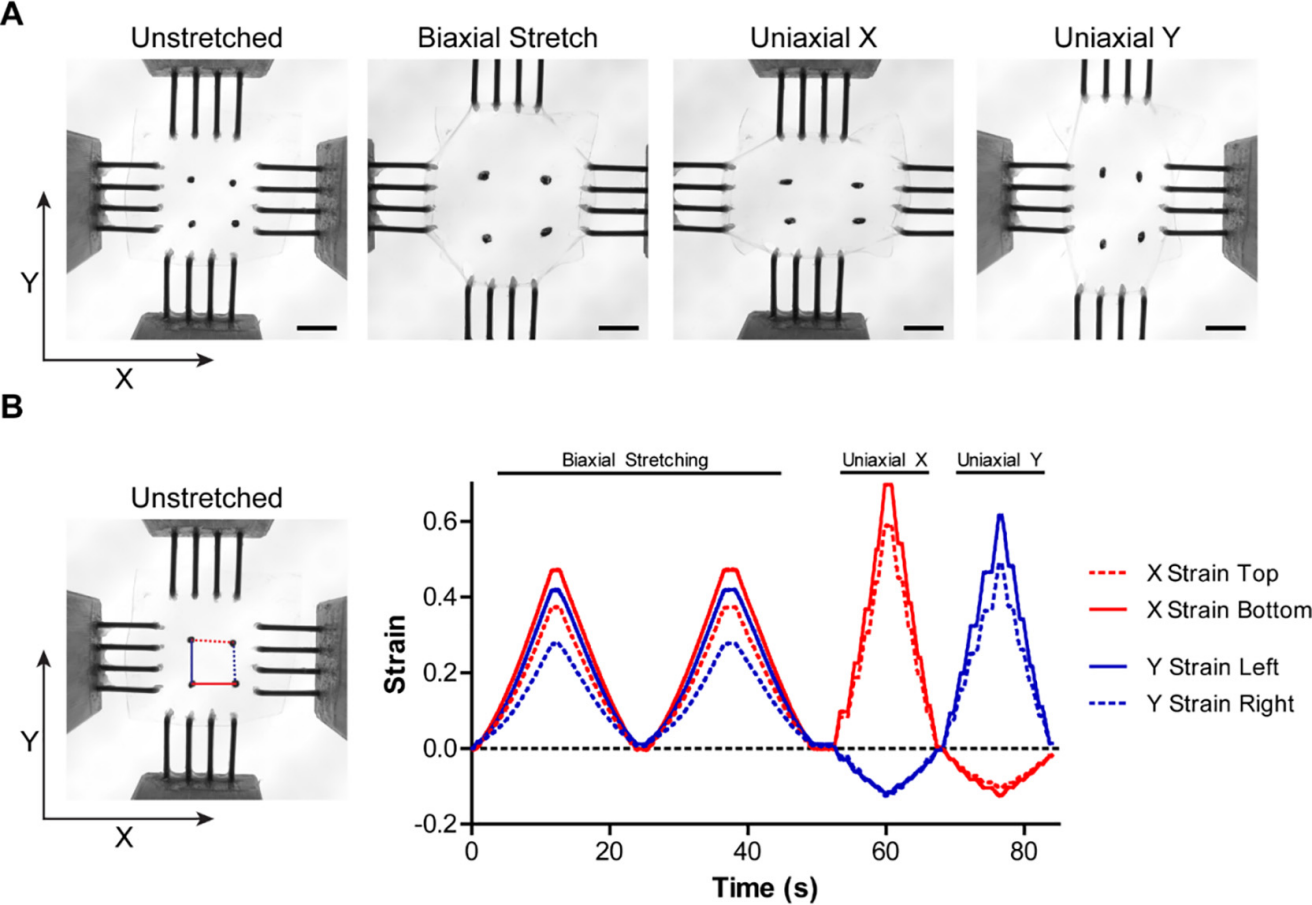
Biaxial and uniaxial stretching of PDMS membrane. (A) PDMS membrane mounted onto the OBS with fiducial marks for displacement visualization and quantification (Scale bars = 5 mm). (B) Point tracking and quantification of the displacement for each fiducial mark shows high repeatability during biaxial and uniaxial stretching (X top = Red dotted, X bottom = Red Solid, Y left = Blue solid, Y right = Blue dotted). (For interpretation of the references to color in this figure legend, the reader is referred to the web version of this article.)

For initial validation and characterization, we chose to perform biaxial and uniaxial tensile testing on a thin film of polydimethylsiloxane (PDMS). Fiducial marks were placed ~5 mm apart in a square orientation on the top surface of the PDMS to track displacement during stretching. Time-lapse videos were acquired to visualize the deformation during tensile testing and for quantitative evaluation. Engineering strain (ε) was calculated by measuring the initial length between two fiducial marks ($l_{0}$) and subtracting it from the observed change in length (l), and then dividing by the initial length ($l_{0}$), $\varepsilon =\left(l-l_{0}\right)/l_{0}$. For each pair of fiducial marks, segment lengths representing the X and Y strain were measured. The OBS was able to perform biaxial stretching and uniaxial stretching in both the X and Y axes (Fig. 22A). During biaxial stretching we demonstrated a user generated strain profile for two stretch and relaxation cycles executed using a g-code macro (Fig. 22B). The macro scripting allows for repeatable control over strain magnitude, duration, and velocity during stretching experiments. In uniaxial stretching along the X and Y axes we demonstrated the ability to incrementally increase strain of the sample and hold at a certain strain using the PanelDue controls (Fig. 22B). This approach is useful in experiments where images are acquired in between strain intervals, or when observing effects following applied strain.

Since the hardware, software, and firmware used for the OBS share specifications with those used in desktop 3D printers, the high precision, repeatability, and motion characteristics are comparable. For the OBS, the 0.9 degree stepper motors utilized allow for a 10 ± 0.5 µm stretch increment, calculated using the Duet WiFi’s 16x microstepping, the 0.9 degree stepper motor’s 5% error per step, and the pully diameter and pitch. For the biaxial stretching experiments, the average deviation in peak strain was 0.068% ± 0.053, suggesting high repeatability between stretch intervals (Fig. 22B, mean ± stdev, Supplemental Video 1). The maximum deviation in parallelism is ~7 milliradians, which for a 5 mm increase in separation between the crossbars amounts to ~40 µm of perpendicular travel. The maximum deviation in perpendicularity is also ~7 milliradians, which produces similar scale errors in travel. Near the center of the sample, where we interrogate strain with confocal microscopy, the errors are much smaller, enabling us to keep a single cell within the field of view. The frequency range possible for cyclic stretch with our biaxial stretching system is limited by the performance of the motors, the distance of travel, and rate of acceleration and deceleration. For example, the maximum frequency possible with a 5 mm displacement defined as an expansion followed by a contraction was approximately 5 Hz (Supplemental Video 2). For other distances the maximum frequency would be slightly different.

Video 1Biaxial stretching of PDMS.
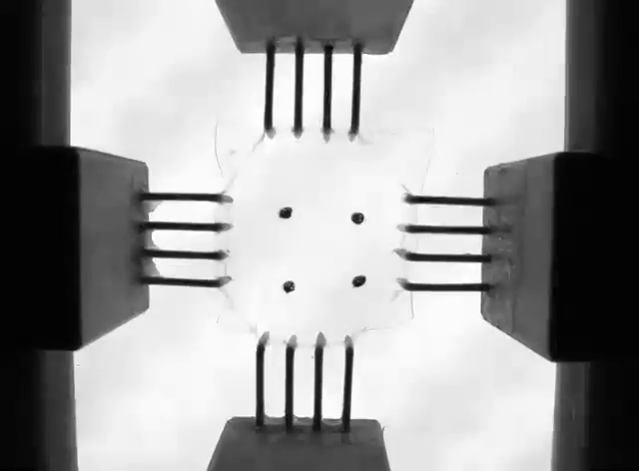

Video 2Maximum cyclic frequency for a 5 mm displacement.
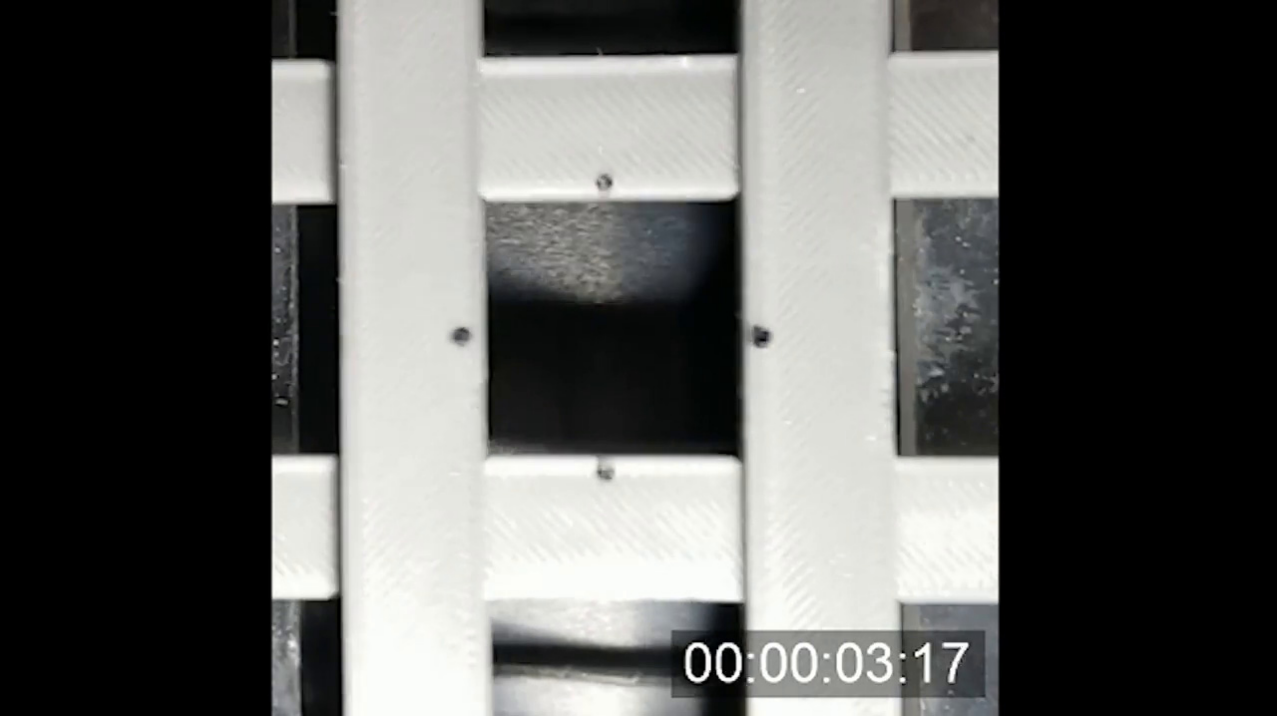

**Fig. 23:**
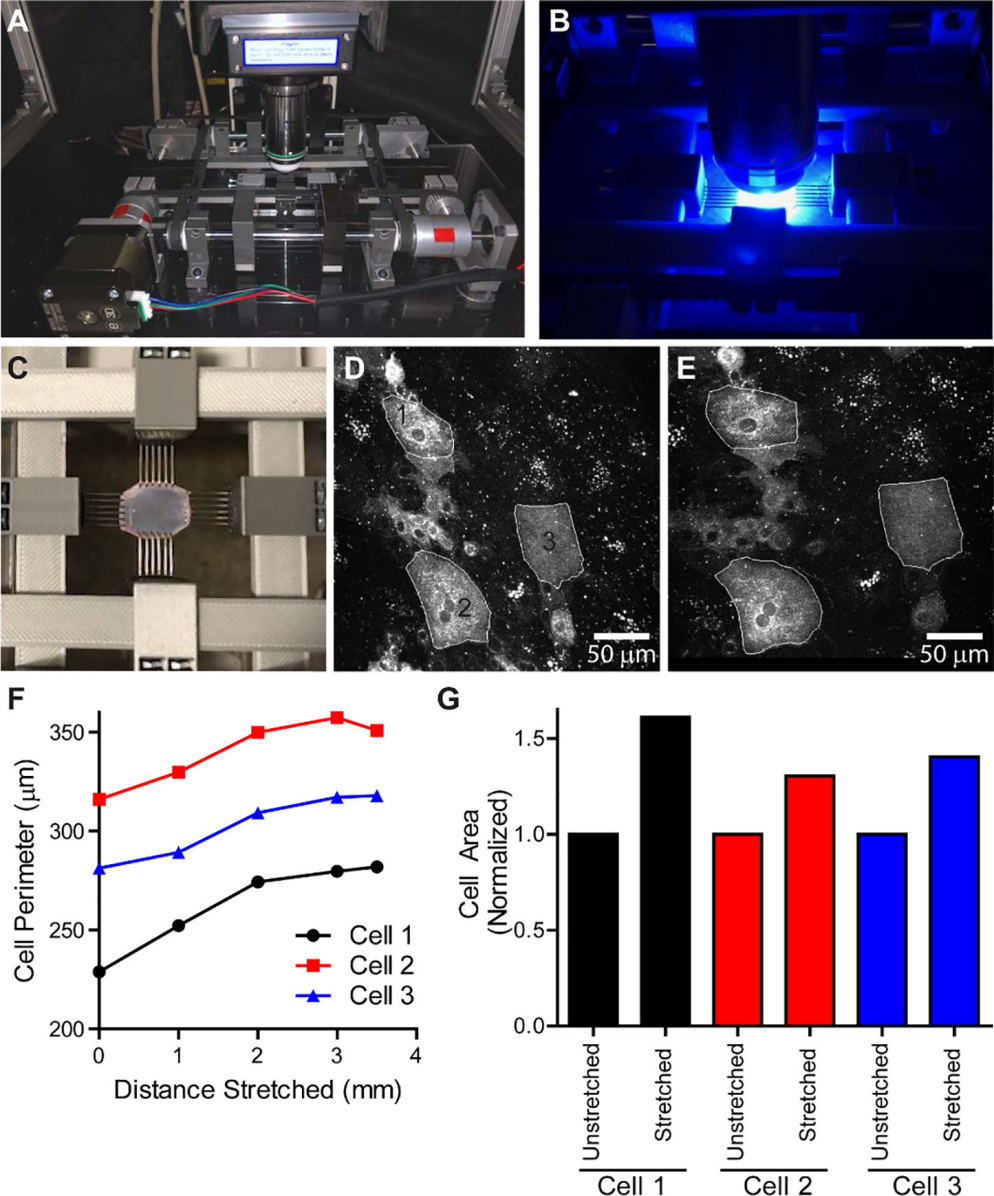
Biaxial Stretching and live fluorescence imaging of umbrella cells in rat bladder. (A) The OBS mounted on a Nikon A1R MP upright confocal microscope stage. (B) Live fluorescence imaging of a bladder sample mounted onto the OBS. (C). Visualization of a suspended rat bladder sample being tensioned by the OBS. (D) Unstretched umbrella cells expressing GFP-Claudin-8. (E) Umbrella cells following 3.5 mm biaxial stretching expressing GFP-Claudin-8. (F) Quantification of the change in cell perimeter during biaxial stretching intervals of 1 mm and 0.5 mm of the cells labeled in panel D. (G) Quantification of the cell area for 3 individual cells before and after 3.5 mm of biaxial stretching.

We next wanted to validate the OBS ability to biaxially stretch a living tissue sample while performing confocal fluorescence microscopy. The tissue we choose to investigate was the urinary bladder. The bladder is a highly elastic tissue and undergoes large expansion and contraction during bladder filling and voiding. For proper function, the bladder cells that make up the apical membrane, umbrella cells, must expand to accommodate the changes in wall tension during bladder filling and voiding [10], [11]. Our goal was to utilize the OBS to biaxially expand bladder tissue to investigate umbrella cell membrane expansion using live cell fluorescence imaging. The OBS was placed onto the stage of a Nikon A1R MP+ upright confocal microscope (Fig. 23A). Rubber pads on the underside of the OBS dampen vibrations and allow for placement onto the microscope stage without the need for additional mounting hardware. The heated bed was set to 42 °C which maintained a sample bath containing a Krebs physiological buffer solution at 37 °C for proper maintenance of the tissue. The temperature of the bath was monitored throughout the experiment with a second thermistor to maintain constant temperature. Rat bladder tissue was isolated and infected with a virus expressing GFP-Claudin-8 to fluorescently label the membrane junctions of urinary bladder umbrella cells following established protocols [10], [11]. The bladder tissue was treated with 50 mM nifedipine for 20 min to inhibit slow wave muscular contractions and mounted onto the OBS via tissue rake attachments. Imaging of GFP-Claudin-8 was used to find a field of view with cells fluorescently labeled (Fig. 23B). The PanelDue 5i manual controls were used to place the bladder under initial tension prior to biaxial stretching (Fig. 23C).

Simultaneous biaxial stretching and live resonant confocal fluorescence imaging was performed using a Nikon 16X 0.8 NA objective with a 3 mm working distance. 3-dimensional Z-stack images were acquired during each stretching interval and then a maximum intensity Z projection was performed to quantify change in cell area and perimeter. Cell perimeters were drawn and measured in ImageJ software to determine the changes in perimeter length and area for each stretching step (Fig. 23D). We performed biaxial stretching using a macro for 1 mm displacements in both X and Y directions (Fig. 23E). Following stretching with the OBS, the average umbrella cell perimeter increased by ~16% and the cell area increased by ~35% (Fig. 23F, and G, Supplemental Video 3). These data validate our previous measurements obtained via fixed cell imaging to confirm that during apical membrane tensioning the urinary umbrella cells expand their membrane to accommodate the increased tension [11]. These results highlight the utility of the OBS to be used in combination with live cell fluorescence imaging to investigate the interplay between biomechanics, cell morphology, and tissue physiology. Future work will utilize this platform to investigate effects of mechanical stretching on urinary bladder, umbrella cell membrane trafficking, and cellular signaling process in various disease states.

Video 3Biaxial stretching and fluorescence imaging of urinary umbrella cells.
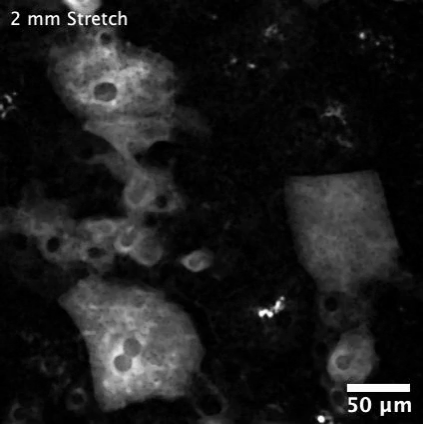

## 8 Human and Animal Rights

Urinary bladders were obtained from female Sprague–Dawley rats (250–300 g; Envigo, Harlan Laboratories, Frederick, MD) according to previously published protocols [11]. All experiments involving animal use and animal tissue were conducted following the National Institutes of Health guide for the care and use of Laboratory animals (NIH Publications No. 8023, revised 1978), and in accordance with guidelines and regulations of the Public Health Service Policy on Humane Care and Use of Laboratory Animals and the Animal Welfare Act under the approval of the University of Pittsburgh Institutional Animal Care and Use Committee.

## Declaration of Competing Interest

The authors declare that they have no known competing financial interests or personal relationships that could have appeared to influence the work reported in this paper.
